# Overexpression of a plasma membrane protein generated broad‐spectrum immunity in soybean

**DOI:** 10.1111/pbi.13479

**Published:** 2020-10-09

**Authors:** Micheline N. Ngaki, Dipak K. Sahoo, Bing Wang, Madan K. Bhattacharyya

**Affiliations:** ^1^ Department of Agronomy Iowa State University Ames IA USA; ^2^ Present address: Department of Energy Joint Genome Institute Walnut Creek CA USA

**Keywords:** PAMP, MAMP, HAMP, pattern recognition receptors, PTI, plasma membrane, soybean sudden death syndrome, soybean cyst nematode, spider mites, soybean aphids, disease resistance, chitin

## Abstract

Plants fight‐off pathogens and pests by manifesting an array of defence responses using their innate immunity mechanisms. Here we report the identification of a novel soybean gene encoding a plasma membrane protein, transcription of which is suppressed following infection with the fungal pathogen, *Fusarium virguliforme*. Overexpression of the protein led to enhanced resistance against not only against *F. virguliforme*, but also against spider mites (*Tetranychus urticae,* Koch), soybean aphids (*Aphis glycines,* Matsumura) and soybean cyst nematode (*Heterodera glycines*). We, therefore, name this protein as *Glycine max disease resistance 1* (*GmDR1; Glyma.10g094800*). The homologues of *GmDR1* have been detected only in legumes, cocoa, jute and cotton. The deduced GmDR1 protein contains 73 amino acids. GmDR1 is predicted to contain an ecto‐ and two transmembrane domains. Transient expression of the green fluorescent protein fused GmDR1 protein in soybean leaves showed that it is a plasma membrane protein. We investigated if chitin, a pathogen‐associated molecular pattern (PAMP), common to all pathogen and pests considered in this study, can significantly enhance defence pathways among the *GmDR1*‐overexpressed transgenic soybean lines. Chitin induces marker genes of the salicylic‐ and jasmonic acid‐mediated defence pathways, but suppresses the defence pathway regulated by ethylene. Chitin induced SA‐ and JA‐regulated defence pathways may be one of the mechanisms involved in generating broad‐spectrum resistance among the *GmDR1*‐overexpressed transgenic soybean lines against two serious pathogens and two pests including spider mites, against which no known resistance genes have been identified in soybean and among the most other crop species.

## Introduction

Food supply is often interrupted severely by devastating plant disease and pest epidemics. One of such major plant disease outbreaks is the Irish famine of 1845–1852 caused by late blight disease in potatoes, in which one million people died from starvation (Griffith, 2007). Plant breeders constantly breed disease and pest‐resistant crop varieties to secure food supply. Plant disease resistance mechanisms are highly complex (Andersen *et al*., 2018; Dodds and Rathjen, 2010). Pattern‐triggered immunity (PTI) activated by pathogen‐associated molecular patterns (PAMPs), herbivore‐associated molecular patterns (HAMPs), nematode‐produced ascarosides or unknown molecular patterns is the first layer of plant defences, which is overcome by pathogen effector proteins to cause effector‐triggered susceptibility (ETS) (Jones and Dangl, 2006). Plants then have evolved with receptors that recognize some of these effectors and trigger a strong form of disease resistance, named effector‐triggered immunity (ETI) (Jones and Dangl, 2006; Zipfel, 2014). Receptors that recognize pathogen effector proteins to induce ETI often contain nucleotide‐binding leucine‐rich repeat (NB‐LRR) domains (Macho and Zipfel, 2014). PTI and ETI defend plants from most pathogen and pest attacks by activating one or more signalling pathways regulated by plant hormones such as salicylic acid (SA), jasmonic acid (JA), abscisic acid (ABA) and ethylene (Bigeard *et al*., 2015; Kunkel and Brooks, 2002).

ETI has been extensively applied in breeding disease‐resistant varieties in most crop species (Gu *et al*., 2005). It’s usually effective only against a subset of a pathogen population and is not broad‐spectrum. In contrast, PTI provides broad‐spectrum and low level or partial resistance, not only against all isolates of a single pathogen, but also against multiple plant pathogens (Bigeard *et al*., 2015).

Plants possess numerous genes that encode putative surface receptors; for example, transmembrane receptor kinases (RKs) (Zipfel, 2014). Many of these genes may have been evolved to regulate plant defences. Unfortunately, the majority of these genes are yet to be studied. Characterized plant pattern recognition receptors (PRRs) involved in PTI are classified into: (i) receptor‐like kinases (RLKs) and (ii) receptor‐like proteins (RLPs) (Macho and Zipfel, 2014). RLPs do not contain a kinase domain as observed in RLKs for signalling. Interaction of an RLP and a kinase with an RLK for stem and floral meristem development has been demonstrated (Bleckmann *et al*., 2010).

Recognition of molecular patterns of plant‐pathogen and pests by plant receptors, PRR, is poorly understood. Bacterial flagellin and peptidoglycans are shown to be the ligands of PRRs (Zipfel, 2004). Nematode‐produced ascarosides have been considered to be recognized by PRRs to signal plant defences (Manosalva *et al*., 2015). Plant PRRs have been shown to recognize insect PAMPs or HAMPs (Gouhier‐Darimont *et al*., 2013; Mithöfer and Boland, 2008; Prince *et al*., 2014). Damage‐associated molecular patterns (DAMPs) caused by infection are recognized by wall‐associated kinases (WAKs) (Choi and Klessig, 2016; Zipfel, 2014).

Plant lysin motif (LysM)‐containing receptors recognize chitin, an elicitor or MAMP for activation defence responses in plants (Dworkin, 2018; Gallego‐Giraldo *et al*., 2018; Khan *et al*., 2003; Sánchez‐Vallet *et al*., 2015; Shi *et al*., 2019; Wan *et al*., 2008a). Chitin is found among a wide range of pathogen and pests including *Fusarium* spp., nematodes, aphids and spider mites (Bos *et al*., 2010; Chen and Peng, 2019; Sánchez‐Vallet *et al*., 2015; Zhou *et al*., 2017).

Worldwide, soybean is an economically very important crop. In the United States, soybean suffers annual yield suppression valued over $5 billion from various pathogenic diseases (Allen *et al*., 2017). Sudden death syndrome (SDS) is one of the most serious soybean diseases, which is caused by the fungal pathogen *Fusarium virguliforme*. The pathogen infects and colonizes soybean roots causing necrosis and root rot, and subsequently foliar SDS, which is characterized initially by leaf chlorosis followed by necrosis, leaf and pod drops. The pathogen remains in roots and releases phytotoxins to cause foliar SDS (Brar and Bhattacharyya, 2012; Brar *et al*., 2011; Pudake *et al*., 2013). In the U.S., recently the yield suppressions from *F. virguliforme* have been reported to be second to that from the most serious pathogen, soybean cyst nematode (SCN). In recent years, the total annual soybean yield suppressions caused by the two pathogens have been valued at close to $2 billion (Allen *et al*., 2017).

In a transcriptomic study of the soybean‐*F. virguliforme* interaction, we observed that the steady state transcript levels of only a few soybean genes were reduced by the *F. virguliforme* infection (Ngaki *et al*., 2016; Sahu *et al*., 2017). One of these genes, *Glycine max disease resistance 1* (*GmDR1*; *Glyma*.*10g094800*) encodes a novel protein with unknown function (Ngaki *et al*., 2016). Overexpression of *GmDR1* in transgenic soybean plants enhances immunity not only against *F. virguliforme*, but also against SCN, spider mites and soybean aphids. GmDR1 is an integral plasma membrane protein. It is predicted to contain an ecto‐ and two transmembrane domains with no kinase domain. The chitin, molecular pattern present in all four pathogen and pests included in this study, induces defence signalling pathways mediated by plant hormones, SA and JA, but suppressed the one mediated by ethylene among the *GmDR1*‐overexpressed soybean plants. We therefore hypothesize that GmDR1 could be a receptor‐like protein. Following ectopic overexpression, it presumably recognizes pathogen and pest‐associated molecular pattern(s) including chitin to initiate the broad‐spectrum disease and pest resistance in soybean.

## Results

### Overexpression of *GmDR1* enhanced *F. virguliforme* resistance

**Figure 1:**
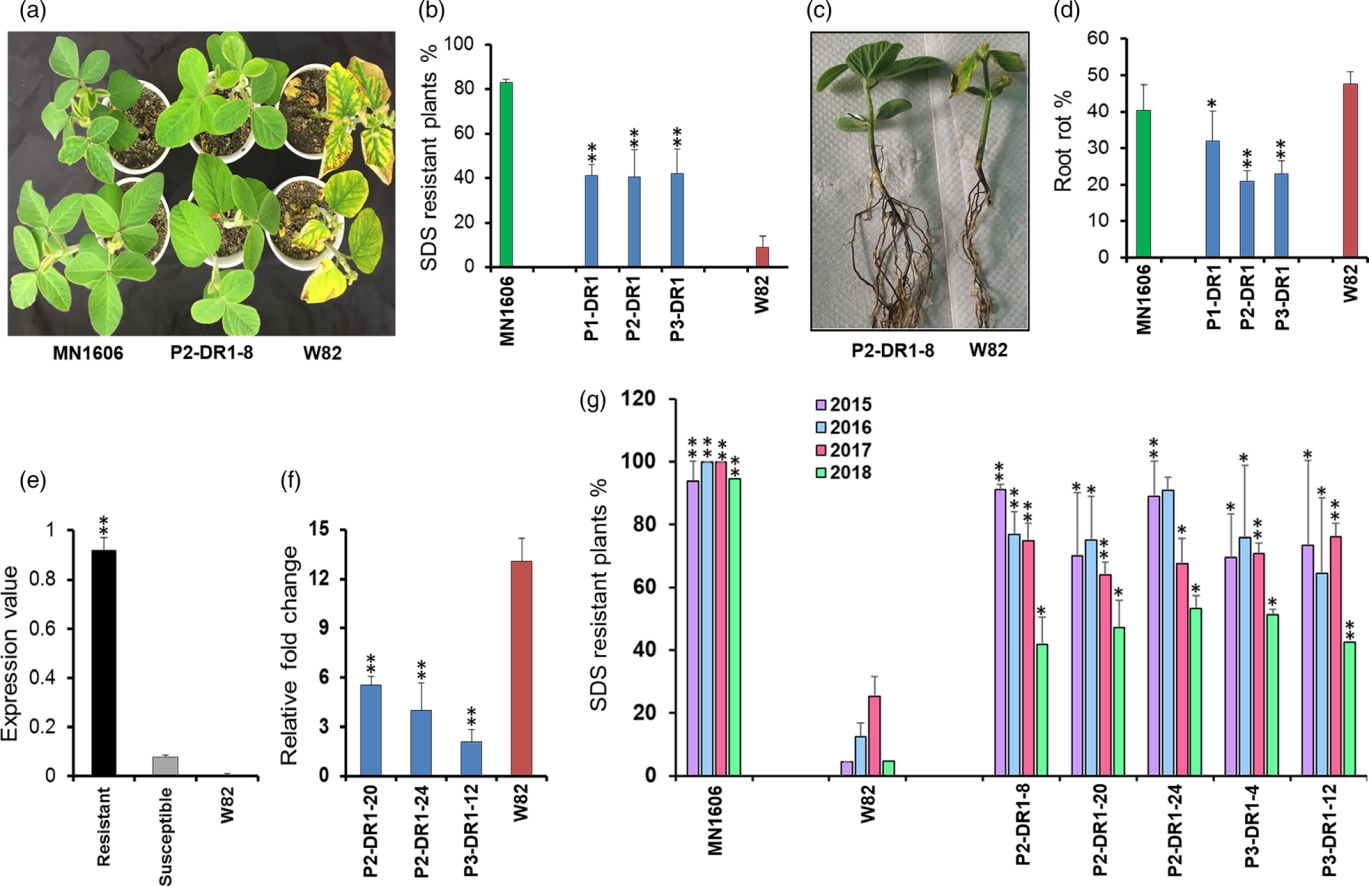
Transgenic soybean plants carrying *GmDR1* transgenes showed enhanced resistance against *F. virguliforme* as compared to the recipient, Williams 82 (W82) line. (a) Enhanced foliar SDS resistance among R_1_ progenies generated from individual *GmDR1* transgenic plants, 4 weeks following root inoculation with the pathogen in a growth chamber. (b) Proportion of R_1_ progenies showing SDS resistance with SDS scores 2 or less. Score 1, no symptoms, Score 2, slight yellowing. Data are means and ± SEs of all R_1_ progenies of individual transgenes presented in Figure S2a. (c) Root phenotypes of an SDS‐resistant R_1_ progeny and Williams 82 shown in (a). (d) Extent of root rot among the resistant and susceptible progenies presented in (a). Data are means and ± SEs of all R_1_ progenies of individual transgenes presented in Figure S2b. Transgenic lines are shown with blue bars, the SDS‐resistant check MN1606 with a green bar, and the SDS susceptible control Williams 82 (W82) with a red bar. (e) Mean *GmDR1* transgene expression levels among SDS‐resistant and susceptible R_1_ progenies presented in Figure S2c. The primers (Table S5) used for RT‐PCR were transgene‐specific; and as expected, no amplification was detected in the nontransgenic Williams 82. (f) Relative biomass of *F. virguliforme* in infected root tissues was calculated as relative amplification of the fungal genomic DNA for the *FvTox1* gene as compared to that for the soybean gene *Glyma.05G014200* in quantitative PCR experiments. Two weeks following infection of roots with *F. virguliforme* or treatment with only water, root tissues were collected for quantitative PCR. Data are averages and ± SEs of three independent experiments, each with 15 plants per line. (g) Foliar SDS resistance among the transgenic soybean lines in 2015, 2016, 2017, and 2018 field trials. Data are means and ± SE of two replications in 2015 and four replications in 2016 and 2017, three replications in 2018, each with 10 to 50 plants. In 2015, 2016, 2017 and 2018 R_1_, R_2_, R_3_ and R_4_ progenies, respectively, were evaluated.

Earlier we have shown that *GmDR1* and a few other soybean genes are down‐regulated following infection with *F. virguliforme* (Ngaki *et al*., 2016). We hypothesized that *F. virguliforme* suppresses the transcription of *GmDR1* to cause susceptibility. To test this hypothesis, we fused *GmDR1* to three infection‐inducible promoters and created three fusion *GmDR1* genes (Figure S1; Tables S1 and S2). A total of 30 independent transformants from these three *GmDR1* fusion genes were generated, and progenies of the transgenic soybean plants were evaluated for responses to *F. virguliforme* infection. It was observed that approximately 40% of the segregating R_1_ progenies of transgenic lines were SDS‐resistant; whereas only 9% of the nontransgenic, *GmDR1*‐fusion gene recipient Williams 82 plants showed SDS resistance (Figure 1a,b; Figure S2a). Furthermore, root rot was significantly reduced among the SDS *F. virguliforme* R_1_ progenies (Figure 1c,d; Figure S2b). *GmDR1* transgenes were expressed among the *F. virguliforme*‐resistant R_1_ progenies; but not among the SDS susceptible progenies (Figure 1e; Figure S2c). No amplification was detected in nontransgenic Williams 82 that did not carry the *GmDR1* transgene (Figure 1e). qPCR of a pathogen and a host gene revealed at least 2.5– to 5‐folds reduction in fungal biomasses in roots of the SDS‐resistant transgenic soybean lines as compared to that in nontransgenic, SDS susceptible Williams 82 line (Figure 1f). The reduced fungal biomasses among the SDS‐resistant transgenic lines was associated with the 2‐fold reduction in root rot symptoms in SDS‐resistant transgenic plants as compared to the nontransgenic Williams 82 plants (Figure 1d).

Transgenic plants carrying the *GmDR1* transgenes were also evaluated for SDS resistance under field conditions. *GmDR1* transgenes enhanced SDS resistance of the transgenic soybean plants during the 2015, 2016, 2017 and 2018 growing seasons. We observed that 65 to 91% of the basta‐resistant transgenic R_1_ plants descended from five independent transgenic R_0_ soybean plants exhibited enhanced SDS resistance under field conditions in 2015 (Figure 1g; Figure S3). The copy number of the R_1_ plants was ascertained by conducting qPCR (Ngaki *et al*., 2016), and seeds of at least one homozygous progeny from individual transgenic lines were planted in the 2016 field trial. Up to 91% of the R_2_ progenies showed SDS resistance with little or no visible foliar SDS symptoms (Figure 1g; Figure S3). Similar results were observed in the 2017 field trial conducted for the R_3_ progenies of the homozygous R_2_ lines (Figure 1g). In 2018, the R_4_ progenies of R_3_ lines were tested against a very high load of the *F. virguliforme*. R_4_ progenies exhibited significantly higher levels of SDS resistance as compared to that in the nontransgenic Williams 82 line (Figure 1g). The responses of the transgenic lines to *F. virguliforme* infection were determined in four independent fields during the 4‐year trial. There were no obvious morphological changes among the transgenic soybean lines. The mean seed size and yield/plant of the transgenic lines were statistically not different from that of the nontransgenic Williams 82 lines (Figure S4). Our data suggest that overexpression of *GmDR1* in roots of transgenic plants enhances resistance against *F. virguliforme* without affecting the yield potential.

### Overexpression of *GmDR1* resulted in induced expression of the *GmPR1‐1* gene, a marker of the defence pathway regulated by SA

**Figure 2:**
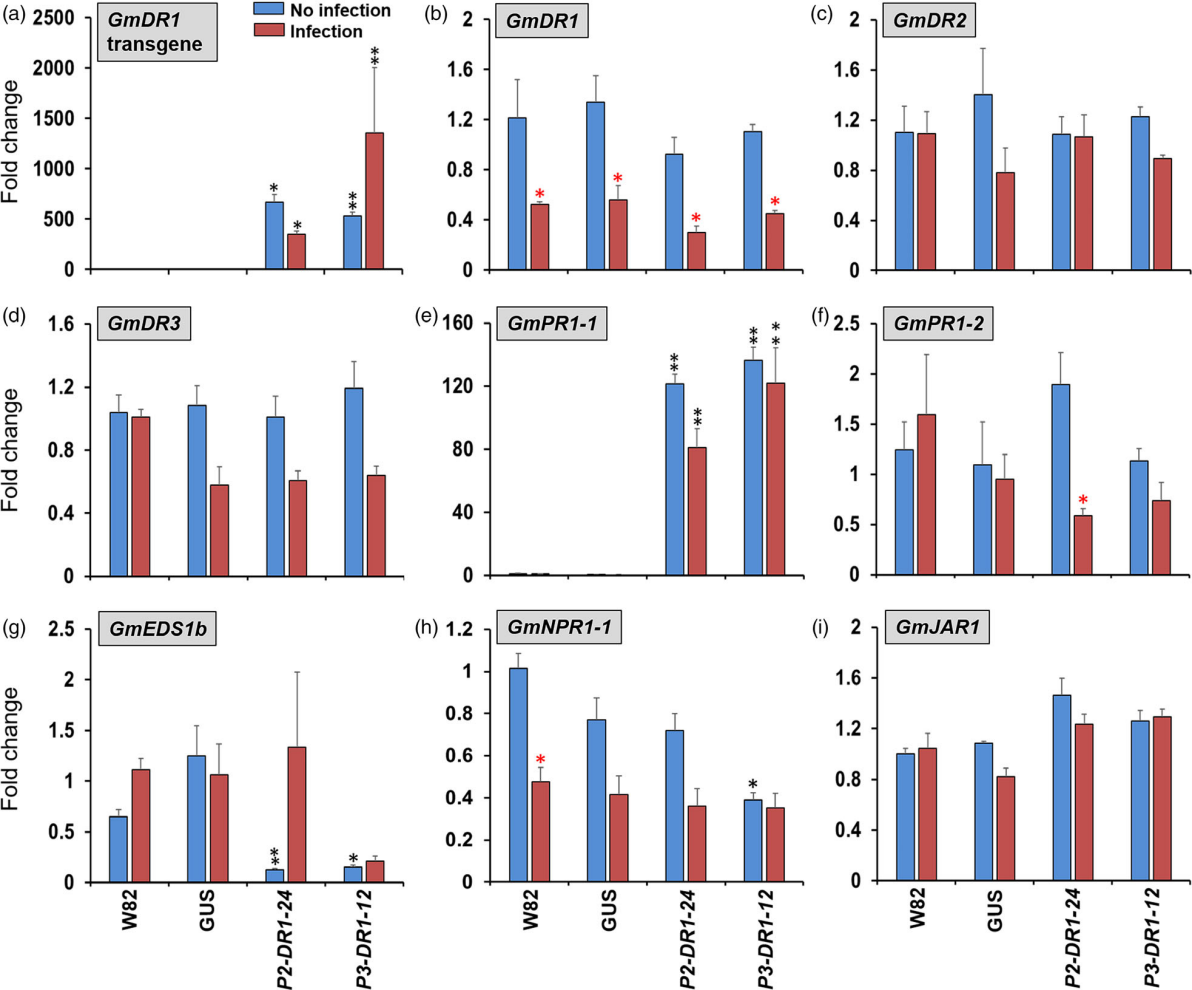
Expression of *GmDR1* transgenes up‐regulates the pathogenesis‐related gene *GmPR1‐1* in roots of *GmDR1* transgenic plants. Relative transcript abundance of (a) *GmDR1* transgenes, (b) *GmDR1* endogenous gene, (c) *GmDR2* (*Glyma.02g180500*), (d) *GmDR3* (*Glyma.03g139900*), (e) *GmPR1‐1* (*Glyma.15g062500*), (f) *GmPR1‐2* (*Glyma.13G251600*), (g) *GmEDS1b* (*Glyma.06g19920*), (h) *GmNPR1‐1* (*Glyma09g02430*), (i) *GmJAR1* (*Glyma.03G256200)* in soybean roots following SDS infection. Data represent mean ± standard error of three independent experiments. Each experiment contains three biological replicates of 6 pooled seedlings for each genotype in each treatment. Expression values were normalized to the expression levels of the constitutively expressed *Elongation factor 1‐b* (*ELF1‐b*) gene (*Glyma.02g44460*) in respective samples. *, significantly different to the control W82; *, significantly different to non‐infected plants for the same genotype. One star, *P* < 0.05; two stars, *P* ≤ 0.001. P1, P2, and P3 are promoter 1, promoter 2, and promoter 3 (Table S1), respectively. W82, Williams 82. GUS, a transgenic line harbouring the *Gus* gene (Gus) in place of the *GmDR1* transgene.

The *GmDR1*‐transgenes were highly expressed among the roots of transgenic lines (Figure 2a). The overall expression levels of *GmDR1‐*transgenes were ~ 500‐folds higher than that of the constitutively expressed soybean *ELF1b* gene. As observed before, the expression of the endogenous *GmDR1* gene was suppressed among the nontransgenic and transgenic soybean plants following *F. virguliforme* infection (Figure 2b; Ngaki *et al*., 2016). The expression of *GmDR1*‐homeologues, *GmDR2* (*Glyma.02g180500.1*) and *GmDR3* (*Glyma.19G142700.1*), was not influenced by *F. virguliforme* infection (Figure 2c,d). The *GmDR4* (*Glyma.03g139900.1*) transcripts were not detected among the roots of the either nontransgenic or transgenic soybean plants (data not presented).

Towards understanding the possible mechanisms of enhanced SDS resistance among the transgenic soybean lines with overexpressed *GmDR1*, we investigated if any of the two major plant hormones, SA‐ and JA‐mediated defence pathways, are altered among the transgenic plants. Transcript levels of the two *GmPR1* homeologues*, GmPR1‐1* and *GmPR1‐2* (Xu *et al*., 2016; Xu *et al*., 2018; Zeng *et al*., 2017), marker genes for the defence pathway regulated by SA, were investigated. Surprisingly, *GmPR1‐1*, but not *GmPR1‐2*, is constitutively induced over 120‐folds more among the transgenic soybean lines as compared to that in the nontransgenic Williams 82 control (Figure 2e,f). The enhanced *GmPR1‐1* transcript levels of the transgenic soybean lines were however not changed following *F. virguliforme* infection. Transcript levels of *GmPR1* genes were also not induced in nontransgenic Williams 82 following inoculation with *F. virguliforme* infection (Figure 2e,f).

Two soybean homologues of the positive basal resistance regulator Arabidopsis *EDS1, GmEDS1a* (*Glyma04g34800*) and *GmEDS1b* (*Glyma.06g19920*), were also investigated for their expression patterns following *F. virguliforme* infection (Wiermer *et al*., 2005). There were no significant differences in the *GmEDS1a* transcript levels between the nontransgenic and transgenic soybean lines (data not shown). However, the expression of *GmEDS1b* was significantly suppressed among the transgenic lines as compared to the control nontransgenic soybean lines (Figure 2g). The expression of *GmNPR1‐1* but not *GmNPR1‐2* was significantly reduced in one of the *GmDR1*‐overexpressed lines (Figure 2h). The expression of *GmNPR1‐1* was also significantly reduced in Williams 82 following *F. virguliforme* infection (Sandhu *et al*., 2009; Figure 2h). Transcript levels of the JA pathway marker, *GmJAR1,* were unchanged (Figure 2i).

### Overexpression of *GmDR1* resulted in novel immunity against the spider mites

Two‐spotted spider mites (*Tetranychus urticae* Koch) are leaf‐feeding pests that cause yellow and brown leaf spots, bronze colour in the entire leaf blade and finally develop mite‐webs leading to severe yield losses (Jimenez, 2014). Unfortunately, no acceptable mite resistance (http://corn.agronomy.wisc.edu/Management/pdfs/A3890.pdf) has been reported in soybean and many other crop species (Agut *et al*., 2018). SA and JA regulate mite infestation of plants (Arena *et al*., 2018).

**Figure 3:**
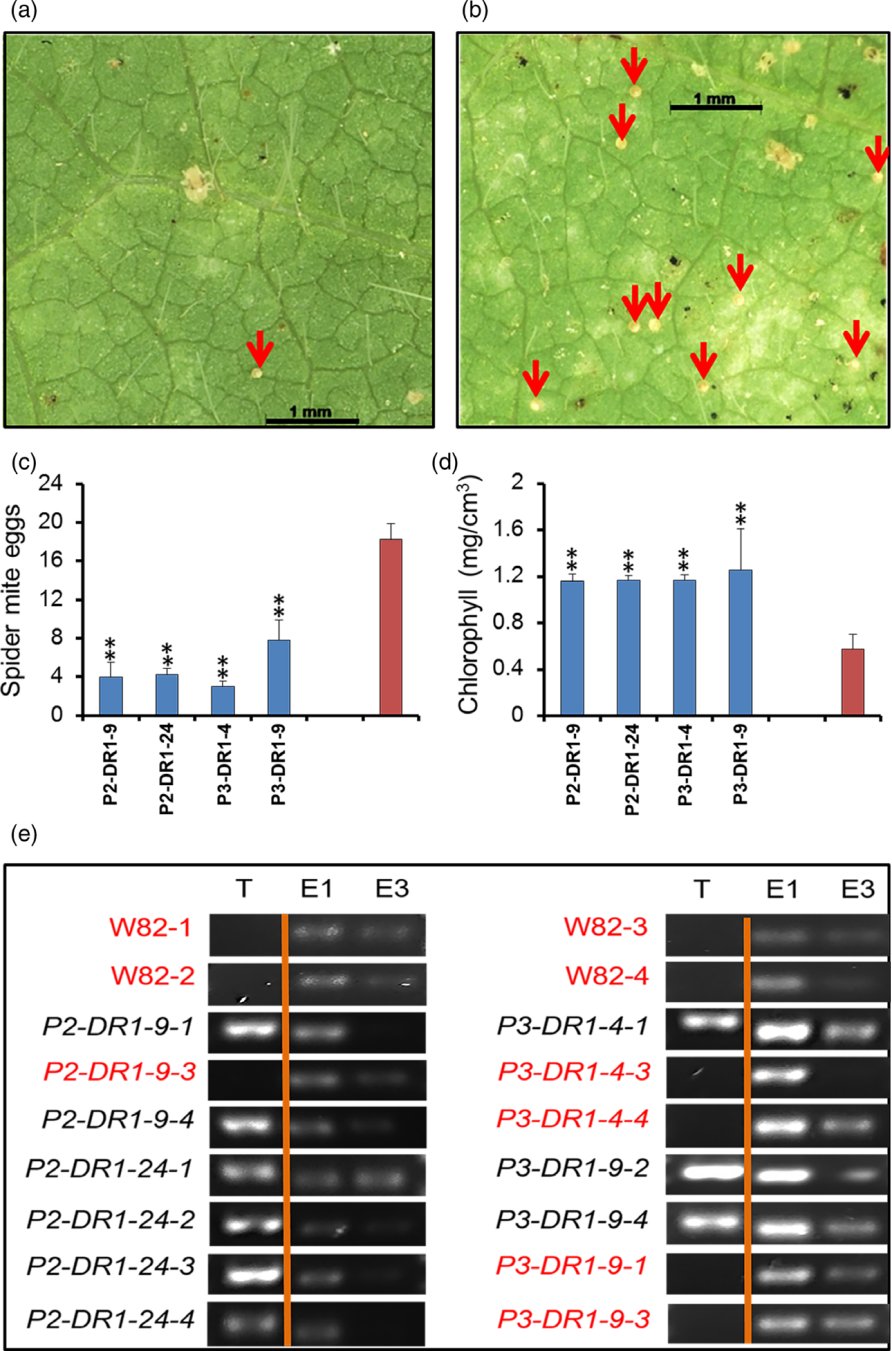
Expression of *GmDR1* conferred immunity of transgenic soybean plants against the soybean spider mites. (a) Leaf blade of an R_1_ transgenic plant carrying the *P2‐DR1* transgene 5 days following spider mite inoculation. Red arrow points to an egg. (b) Leaf blade of the nontransgenic Williams 82 line 5 days following inoculation. (c) Spider mite egg numbers of individual R_1_ progenies 5 days following inoculation with 40 adult mites (Figure S5‐6). (d) Chlorophyll contents of the mite‐infested leaf blades. (e) Expression of *GmDR1* transgenes (T) and the endogenous *GmDR1* gene (E1 and E3). E1 and E3, RT‐PCR products of the endogenous *GmDR1* gene one‐ and 3‐day following mite inoculation. Genotypes shown with red and black font colours indicate mite‐susceptible and mite‐resistant plants respectively. W82, Williams 82.

In February of 2014, spider mites infested severely all R_0_ transgenic plants except the ones that carried the *P2‐DS1* transgene, grown in the greenhouse (Figure S5a). Some of the R_1_ progenies of the transgenic plants carrying *GmDR1* transgenes defended mite infestation under greenhouse conditions (Figure S5b‐d). Significantly reduced number of mite‐eggs was observed on leaf blades of the *GmDR1*‐transgenic plants as compared to that on the leaf blades of nontransgenic Williams 82 plants (Figure 3a‐c; Figure S6). Leaves of the mite‐resistant transgenic plants have shown to sustain higher chlorophyll contents as compared to that in the susceptible, nontransgenic Williams 82 line following mite infestation (Figure 3d). Overexpression of the *GmDR1* transgenes was associated with enhanced mite resistance among the transgenic soybean plants (Figure 3e). Expression of the endogenous *GmDR1* gene was suppressed following mite infestations (Figure 3e).

### Overexpression of *GmDR1* resulted in enhanced immunity against soybean aphids

**Figure 4:**
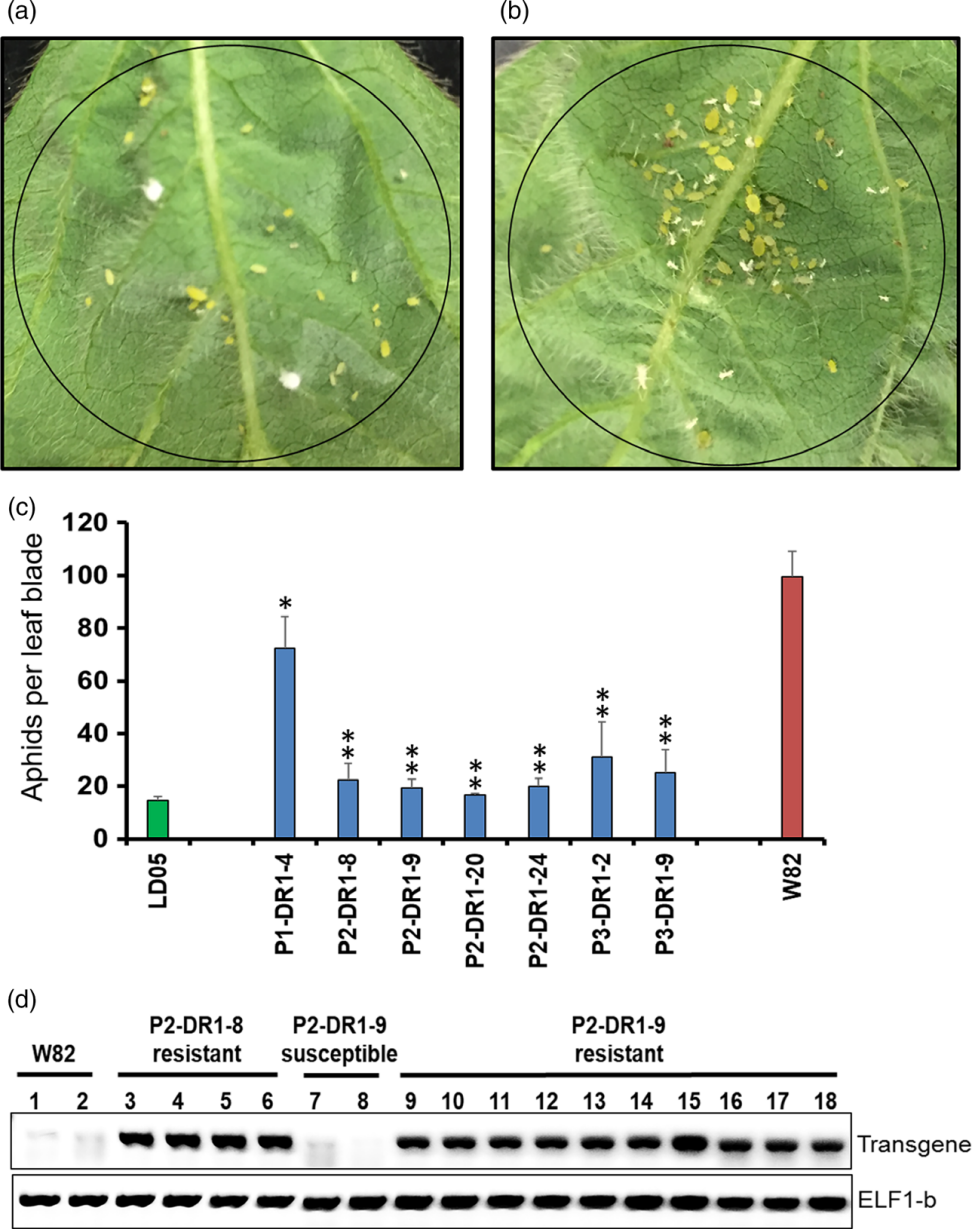
Expression of *GmDR1* conferred immunity of transgenic soybean plants against the soybean aphids. (a) Development of nymphs 7 days following inoculation of a leaf blade of a soybean line carrying the *P2‐GmDR1* transgene with 10 soybean aphids. (b) Development of nymphs 7 days following inoculation of a leaf blade of the nontransgenic Williams 82 line with 10 soybean aphids. (c) Numbers of soybean aphid progenies 7 days following inoculation of leaf blades with 10 soybean aphids. Mean and ± S.E. of aphid incidence among the aphid‐resistant R_1_ progenies (Figure S7b). LD05, an aphid‐resistant LD05‐16060 soybean line; W82, Williams 82. (d) Expression of a *GsDR1‐*transgene in two independent transgenic lines. *ELF‐1b* is the internal control. Significant differences observed between the transgenic and Williams 82 lines are shown with * for *P* < 0.05 and ** for *P* < 0.01. P1, P2 and P3 are promoter 1, promoter 2 and promoter 3, respectively (Table S1).

The soybean aphid (*Aphis glycines* Matsumura) is a sap‐sucking pest. It is a major yield‐reducing pest of soybean (Ragsdale *et al*., 2007). It can damage soybean plants either by feeding on tissues or through transmitting pathogenic viruses (Clark and Perry, 2002). Resistance to aphids is encoded by quantitative trait loci (QTL) as well as single genes (Wiarda *et al*., 2012). Growing aphid‐resistant cultivars is the most effective method of controlling this pest (Hesler *et al*., 2013). We investigated if overexpression of the *GmDR1* transgenes enhanced aphid resistance among the transgenic soybean plants. The progenies of six independent transgenic soybean plants, generated from three independent *GmDR1* transgenes, were investigated for responses to aphid infestation in clip‐cage experiments (Myers and Gratton, 2006). The number of aphids on the leaves of transgenic lines was up to 5‐fold less that on the leaves of nontransgenic Williams 82 plants (Figure 4a‐c; Figure S7). The expression of soybean aphid resistance was associated with the expression of *GmDR1‐*transgenes among the transgenic lines (Figure 4d).

### Overexpression of *GmDR1* resulted in enhanced immunity against the soybean cyst nematode

**Figure 5:**
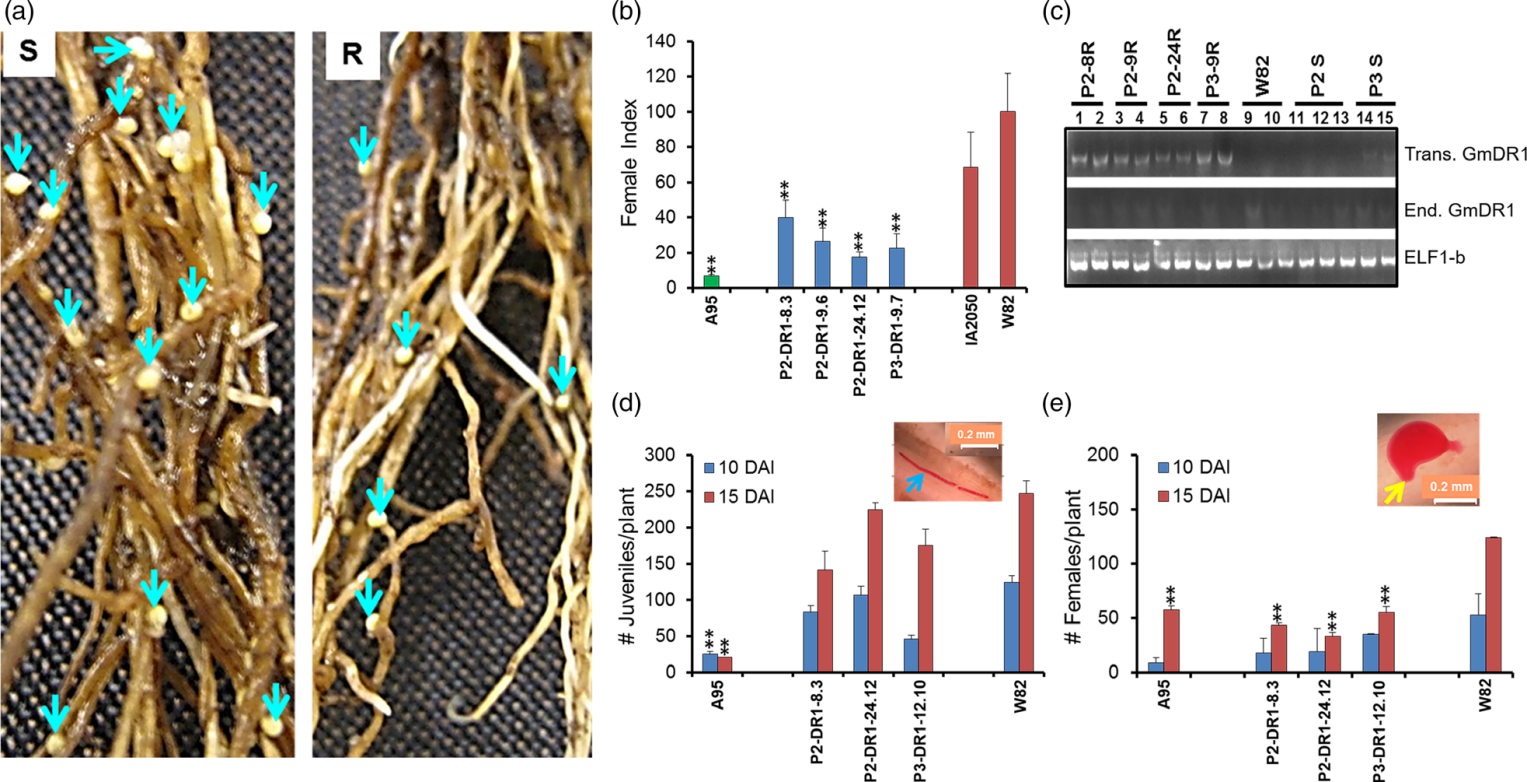
Transgenic soybean plants carrying *GmDR1* transgenes showed enhanced resistance against *H. glycines* as compared to the recipient, Williams 82 (W82) line. (a) Roots of an SCN susceptible plant (left panel) and a transgenic‐resistant plant (right panel) 4 weeks following inoculation with the SCN HG Type 2.5.7. Arrows show the cysts, each containing innumerable number of eggs. Each cyst is developed from a single female nematode. (b) Female indices of individual R_3_ soybean lines. Transgenic lines are shown with blue bars, the resistant check A95‐684043 (A95) with a green bar, and the SCN susceptible Williams 82 and IA2050 cultivars are shown with red bars. Data are averages and ± SEs of three independent experiments with six plants per line in each experiment. (c) Expression of *GmDR1* transgenes among the R_1_ progenies. *ELF‐1b* (*Glyma02g44460*) is the internal control. Lanes 1 to 8 are for SCN‐resistant plants and rest are for SCN susceptible lines. (d) Average number of juveniles per plant, 10 and 15 days after inoculation (DAI). A juvenile is shown with a blue arrow. (e) Average number of females per plant, 10 and 15 DAI. A female is shown with a yellow arrow. Data are averages and ± SEs of means of six plants evaluated in each of the three independent experiments. Significant differences observed between the transgenic and nontransgenic Williams 82 plants with *, *P* < 0.05 and **, *P* < 0.01. P1, P2 and P3 are promoter 1, promoter 2 and promoter 3; W82 is Williams 82.

The soybean cyst nematode (SCN; *Heterodera glycines*) is a root‐feeding parasite. In the U.S., it is the most serious soybean pathogen that causes annual yield suppression valued at over $1 billion (Allen *et al*., 2017; Mitchum *et al*., 2012). SCN resistance genes are deployed worldwide in breeding SCN‐resistant soybean cultivars to reduce the crop losses from this serious pathogen (Guo X *et al*., 2015; Liu *et al*., 2012). We investigated if the transgenic plants overexpressing the *GmDR1* transgenes can provide any enhanced SCN resistance. We observed that several R_1_ and R_2_ progenies of independent transformants showed enhanced SCN resistance (Figure 5a). The female indices (FI) were significantly reduced among the transgenic lines as compared to the nontransgenic Williams 82 plants (Figure 5b; Figure S8). Transgenic lines are moderately resistant or moderately susceptible (FI ranged from 11 to 60) (Adee and Johnson, 2008), whereas the transgenes recipient nontransgenic Williams 82 line is highly SCN susceptible. In the SCN‐resistant A95‐684043 line, the FI was < 10. Enhanced SCN resistance among the transgenic lines was associated with the expression of the *GmDR1* transgenes (Figure 5c). The numbers of adult females were significantly lower in the roots of transgenic soybean plants as compared to that in roots of nontransgenic Williams 82 plants, although similar numbers of juveniles were observed among the transgenic and nontransgenic soybean lines (Figure 5d, e; Figure S9).

### Promoters 2 and 3 fused *GmDR1* transgenes are strongly expressed in leaves inducing up‐regulation of the *Aphid‐inducible 1* gene

Promoter 2 and Promoter 3 used in generating the *GmDR1* fusion genes for this study were isolated, respectively, from the *Glyma.10g168900* and *Glyma.20g220800* genes encoding germin‐like proteins (GLPs) of the subfamily 1 member 10 that contain the cupin domain (Lanubile *et al*., 2015). In general, plant GLPs are differentially expressed during plant growth and development. They are responsive to biotic and abiotic stresses including bacteria, fungi, insects, nematodes, salinity, temperature, drought, nutrient, (Davidson *et al*., 2009; Dunwell *et al*., 2008; Gunadi *et al*., 2016; Lanubile *et al*., 2015; Lu *et al*., 2010; Ngaki *et al*., 2016; Wei *et al*., 1998).

In this study, we observed that Promoter 2 (P2) and Promoter 3 (P3) fused *GmDR1* transgenes (*P2‐DS1* and *P3‐DS1*) enhanced SDS and SCN resistance in roots, and spider mite and soybean aphid resistance in leaves of transgenic soybean plants (Figures 1, 3, 4, 5). The two promoters are root‐specific (Gunadi *et al*., 2016). Promoter 3 has the highest expression level in hairy roots, three times more than that of the CaMV 35S promoter. Promoter 2 showed slightly lower activity than Promoter 3 (Hernandez‐Garcia *et al*., 2010). The Promoter 3 is weakly active in leaves (Table S1).

We investigated the expression levels of the *Glyma.10g168900* and *Glyma.20g220800* genes in leaves and root (Figure S10). The two promoters, fused to *GmDR1,* were active in leaves of transgenic soybean lines (Figure S11). There are three additional *GmDR1*‐like genes in the soybean genome (Table S2; Figure S12). Overexpression of *GmDR1* transgenes (Figure S13a) did not significantly influence the expression of endogenous *GmDR1, GmDR2* or *GmDR3* genes (Figure S13b‐d).

In a transcriptomic study of the soybean‐soybean aphid interactions, it was reported that transcripts of only one gene (*Glyma06g14090*) was induced 7 days following aphid infestation (Studham and MacIntosh, 2013). We name the gene as *Aphid‐inducible 1* (*GmAI1*). Considering the induction of aphid and mite resistance among the transgenic soybean lines among the *GmDR1‐*overexpressed transgenic plants, we investigated if the expression of the *GmAI1* gene is influenced by the overexpressed *GmDR1* transgenes in soybean leaves. The expression levels of *GmAI1* were highly increased in both transgenic lines with overexpressed‐*GmDR1* (Figure S13e).

### GmDR1 an integral plasma membrane protein

**Figure 6:**
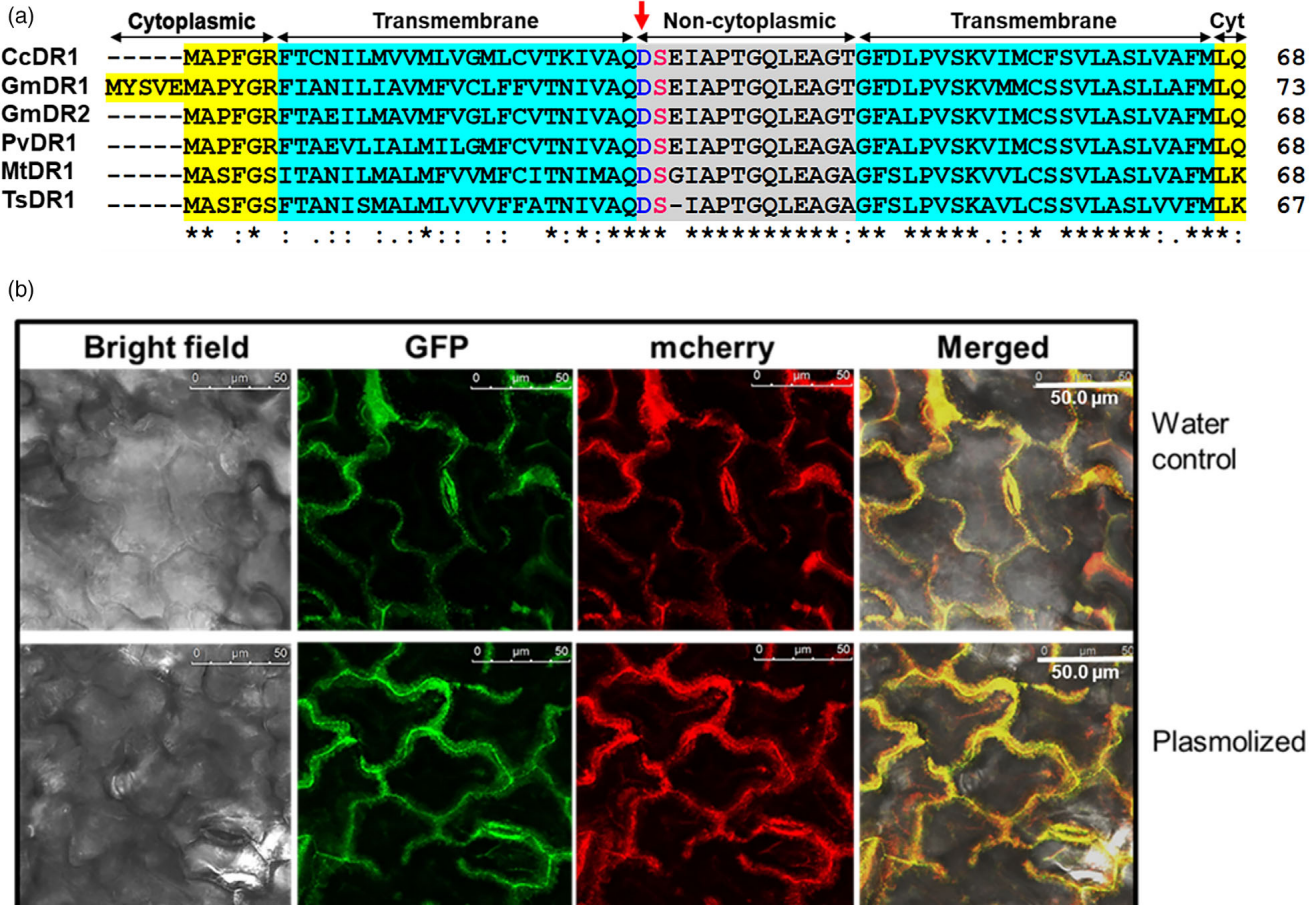
GmDR1 is an integral plasma membrane legume‐specific protein. (a) The putative structure and domains are conserved among the GmDR1 homologs. Yellow, putative cytoplasmic (cyt) domains at N‐ and C‐termini; blue, transmembrane domains; grey, non‐cytoplasmic or ecto‐domain; *, conserved residue; conservative residues that show conservation with groups of amino acids exhibiting strong similar biochemical properties; and, semiconservative residues that show conservation with groups of amino acids exhibiting weak similar properties. D35 (blue font) and S36 (red font) residues are predicted to be involved in protease cleavage and phosphorylation, respectively. Red arrow shows the predicted protease cleavage site. Gm, *Glycine max*; Cc, *Cajanus cajan*; Mt, *Medicago truncatula*; Ts, *Trifolium subterraneum*; Pv, *Phaseolus vulgaris*. (b) Transient expression of green fluorescence protein (GFP) fused GmDR1 (GmDR1‐GFP) and plasma membrane (PM) marker AtPIP2A protein tagged with mCherry (AtPIP2A‐MCH) in *Glycine max* leaf epidermal cells. Confocal microscopy was conducted 72 h following transient co‐expression of GmDR1‐GFP and AtPIP2A‐MCH proteins. Top panel, leaf treated with water droplets. Bottom panel, leaf treated with 5M NaCl droplets. The peripheral distribution of the green and red fluorescence suggests co‐localization of the two transiently expressed fusion proteins in the plasma membrane. Similar results from transient co‐expression of GmDR1‐GFP and AtPIP2A‐MCH proteins in *Nicotiana benthamiana* were observed (Figure S15).

*GmDR1*‐like sequences were detected only in cotton, cocoa, jute and legumes. Phylogenetic analysis of the GmDR1 and its homo‐ and homeologues revealed three clades (Table S3; Figure S12a) with GmDR1 and its three homeologues clustered in two sub‐clades. Alignment of the closely related six GmDR1‐like legume proteins including GmDR1 showed strong conservation of several amino acid residues, of which D35 and S36 residues could be involved in protease cleavage and phosphorylation, respectively (Figure 6a). The sizes of GmDR1 and its homologues range from 67 to 73 aa (Figure 6a).

Functional data indicate that GmDR1 could be a pattern recognition receptor (PRR) that recognizes a molecular pattern common to a variety of organisms including fungus, nematode and insects to induce broad‐spectrum basal plant immunity in transgenic soybean plants (Figures 1, 2, 3, 4, 5). We therefore investigated if it is a plasma membrane bound protein. The 73 aa GmDR1 protein is predicted to have an N‐terminal cytoplasmic domain (11 aa) followed by a transmembrane domain (23 aa), an ecto‐domain (14 aa), a second transmembrane domain (23 aa), and a short cytoplasmic tail (2 aa) (Figure 6a; Figure S14a). The predicted 3D model for the GmDR1 protein revealed two helical regions that perfectly match the predicted transmembrane domains with high confidence (Figure S14b) (Yang and Zhang, 2015).

To experimentally verify its possible plasma membrane residence, subcellular localization study was conducted in *Glycine max* and *Nicotiana benthamiana*. Transient expression of GmDR1 fusion proteins with the GFP tag at its either N‐ or C‐terminus revealed that GmDR1 is localized to plasma membrane (Figure 6b; Figure S15). Investigation of genes co‐expressed with *GmDR1* revealed that many of the co‐expressed genes are involved in cell wall biogenesis and are membrane bound (Table S4).

### Chitin induces SA‐ and JA‐mediated defence pathways among the *GmDR1* transgenic lines

Chitin, a well‐known PAMP or MAMP, is present in *F. virguliforme*, SCN, aphids and spider mites (Bos *et al*., 2010; Chen and Peng, 2019; Sánchez‐Vallet *et al*., 2015; Zhou *et al*., 2017). Considering GmDR1’s plasma membrane residence, we hypothesize that following either direct or indirect interaction with chitin, GmDR1 triggers broad‐spectrum immunity mechanisms against all four pathogen and pests considered in this study. To test this hypothesis, stem‐cuts of 2‐week‐old (i) transgenic plants carrying either *GmDR1* or *GUS* transgene, and ii) transgene‐recipient Williams 82 plants were treated with chitin (Khan *et al*., 2003). qRT‐PCR was conducted to monitor the expression of eight defence genes representing markers of the SA‐, JA‐ and ethylene‐mediated defence signalling pathways. The genes considered were two *GmPR1* genes, *GmPR2*, *GmEDS1*, stress‐induced *NAC transcription factor 6* (*GmNAC6*) gene (*Glyma.12G022700*) and the isochorismate synthase gene *GmICS1* (*Glyma01g25690*) as markers for the SA pathway, *GmJAR1* as the JA pathway marker and the aminocyclopropane‐1‐carboxylate synthase encoded by *GmACS1k* as a marker for the ethylene pathway (*Glyma.16G032200*) (Garcion *et al*., 2008; Lin *et al*., 2013; Melo *et al*., 2018; Pimenta *et al*., 2016; Xu *et al*., 2018).

**Figure 7:**
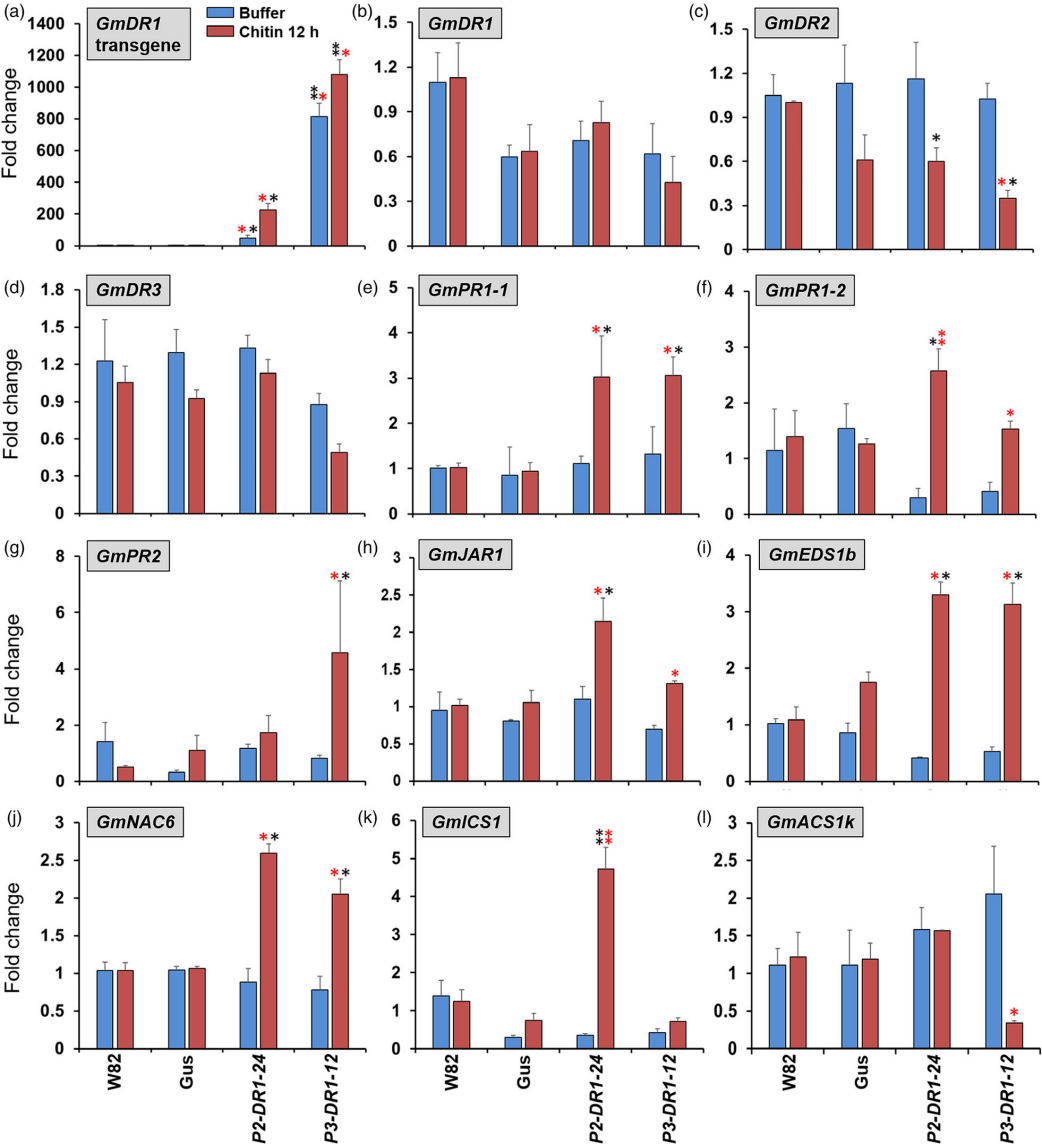
Regulation of defence‐related genes 12 h following treatment with chitin. Relative transcript abundance of (a) *GmDR1* transgenes, (b) endogenous *GmDR1* gene, (c) *GmDR2* (*Glyma.02g180500.1*), (d) *GmDR3 (Glyma.03g139900.1)*, (e) *GmPR1‐1* (*Glyma.15g062500*), (f) *GmPR1‐2* (*Glyma.13G251600*), (g) *GmPR2* (*Glyma.03G132700*), (h) *GmEDS1b* (*Glyma.06g19920*), (i) *GmNAC6* (*Glyma.12G022700*), (j) *GmICS1* (*Glyma01g25690*), (k) *GmJAR1* (*Glyma.19G254000*), (l) *GmACS1k* (*Glyma.16G032200*) 12 h following chitin treatment. Data are mean expression values normalized to the expression levels of the soybean *Elongation factor 1‐b* (*ELF1‐b*; *Glyma.02g44460*) in respective samples and ± standard errors calculated from three independent experiments. In each experiment, three pools of six seedlings for each genotype was considered. *, significantly different to the control W82; *, significantly different due to chitin treatment in a genotype. * or *, *P* < 0.05; ** or **, *P* ≤ 0.001. P2, and P3 are promoter 2 and promoter 3 (Table S1), respectively. W82, Williams 82; GUS, a transgenic line harbouring the *GUS* transgene.

Chitin significantly induced the expression of the two *GmDR1* transgenes but not the endogenous *GmDR1* and *GmDR3* genes (Figure 7a,b,d). The expression of *GmDR4* was not detectible either in the control or in the chitin treated plants (data not presented). The expression of the endogenous *GmDR2* gene was however significantly reduced among the *GmDR1* transgenic lines 12 h following chitin treatment (Figure 7c). Expression of the SA and JA pathway markers were induced at least in one of the two transgenic lines carrying *GmDR1* transgenes (Figure 7e‐k), whereas the expression of the marker for the ethylene pathway was suppressed in one of the two transgenic lines (Figure 7l). No effect of the chitin treatment was observed in the expression of the selected marker genes among the transgenic line carrying *GUS* gene and transgene‐recipient, nontransgenic Williams 82 line.

## Discussion

Four classes of soybean genes including *GmDR1* are down‐regulated following *F. virguliforme* infection (Ngaki *et al*., 2016). *F. virguliforme* presumably manipulates the expression of a few putative defence‐related soybean genes to cause SDS. Exchange of promoters of *GmDR1* and one member each from two other classes of soybean genes with infection‐inducible and strong root‐specific promoters enhanced resistance of transgenic soybean lines to the fungal pathogen *F. virguliforme* (Figure 1; Ngaki *et al*., 2016; M. Ngaki and M.K. Bhattacharyya, unpublished). These results suggest that *F. virguliforme* somehow down‐regulates expression of a few soybean genes to suppress the active defence mechanisms. It could be possible that *F. virguliforme* pathogenicity factors directly bind promoters of defence genes to induce susceptibility. Binding of plant promoters by pathogen effector proteins has been shown to induce susceptibility. It has been concluded that the CRN effector PsCRN108 of the soybean pathogen *Phytophthora sojae* containing a putative DNA‐binding helix‐hairpin‐helix (HhH) motif could be involved in suppressing the expression of plant defence‐related genes by directly targeting specific plant promoters (Song *et al*., 2015).

We have identified three homeologues of *GmDR1*; viz., *GmDR2*, *GmDR3*, and *GmDR4* (Figure S12). The qRT‐PCR results showed that the suppression of transcripts following *F. virguiforme* infection was statistically significant only for the endogenous *GmDR1* gene, but not for the *GmDR1* homologues (Figure 2b‐d). Overexpression of the *GmDR1* gene through swapping its promoter with those of the two root‐specific and *F. virguliforme* infection‐inducible genes led to enhanced resistance of transgenic soybean plants against not only the fungal pathogen *F. virguliforme*, but also against a nematode pathogen, SCN, and two pests, spider mites and soybean aphids, all of which are major deterrents of soybean production (Figures 1, 3, 4, 5; Tables S1‐2; Allen *et al*., 2017; Ngaki *et al*., 2016; Sahu *et al*., 2017; Brandenburg and Kennedy, 1987; Costamagna *et al*., 2007).

Overexpression of *GmDR1* transgenes induced constitutive overexpression of *GmPR1‐1* (*Glyma.15g062500*), but not *GmPR1‐2* (*Glyma.13G251600*), in roots of transgenic lines (Figure 2e,f). Earlier studies have showed that overexpression of *PR1* enhances resistance against both bacterial and fungal pathogens (Alexander *et al*., 1993; Breen *et al*., 2017; Sarowar *et al*., 2005; Shin *et al*., 2014) suggesting the role of antimicrobial activities of the PR1 proteins in plant defences (Boccardo *et al*., 2019; Chen *et al*., 2014; Leah *et al*., 1991; Selitrennikoff, 2001). It is also known that PR1 proteins interact with pathogen effectors (Breen *et al*., 2017; Breen *et al*., 2016; Lu *et al*., 2014). The elevated constitutive expression of *GmPR1‐1* in *GmDR1*‐overexpressed plants is most likely played a major role in enhancing SDS resistance among the transgenic lines (Figure 1).

Williams 82 is an SDS susceptible soybean cultivar. We failed to detect expression of *PR1* genes in roots of Williams 82 following infection with *F. virguliforme* (Figure 2e,f). Earlier, we had failed to observe expression of *GmPR1* gene 3‐ and 5‐day following inoculation of etiolated Williams 82 seedlings with *F. virguliforme* (Ngaki *et al*., 2016). Induction was observed only after 10 days following inoculation. In soybean, transcript levels of *GmPR1‐like* and *GmPR* genes may either decrease or increase in the susceptible soybean lines following infection (Abdelsamad *et al*., 2019; Kim *et al*., 2011).

The transcript levels of *GmEDS1b* were down‐regulated in both *GmDR1*‐overexpressed plants (Figure 2g). EDS1 is a positive regulator of basal resistance in Arabidopsis. Pathogen effectors alters its interaction with receptors that regulate immunity (Bhattacharjee *et al*., 2011). In soybean, EDS1 homologues function however differently as compared to that by Arabidopsis EDS1. Interaction of GmEDS1a/GmEDS1b proteins with the cognate bacterial effector protein is required for virulence function of a bacterial pathogen in soybean (Wang *et al*., 2014). Thus, *EDS1b* expression may have a similar role in pathogenicity function of *F. virguliforme,* and down‐regulation of *EDS1b* in *GmDR1*‐overexpressed plants therefore may contribute towards enhancing SDS resistance.

Like the expression of *GmEDS1b*, the expression of *GmNPR1‐1* was also significantly reduced in one of the *GmDR1*‐overexpressed lines (Figure 2h). The expression of *GmNPR1‐1* was significantly reduced in Williams 82 following *F. virguliforme* infection. It appears that as in *GmEDS1*, *GmNPR1* may also act differently in soybean as compared to that by *NPR1* in Arabidopsis. In wheat, constitutive expression of the positive regulator of immunity Arabidopsis *NPR1* resulted in increased susceptibility to *Fusarium asiaticum* that causes fusarium seedling and head blights (Gao *et al*., 2013). It is becoming apparent that the knowledge gained in the model plant Arabidopsis may not always be translated to all crop species. The resources created in this study should be useful in dissecting immunity signalling pathways in soybean. Our results suggest that SA‐mediated defence signalling pathway could be one of the mechanisms used by *GmDR1* in enhancing immunity of transgenic soybean plants against *F. virguliform*e.

Transcript levels of the constitutively expressed soybean *ELF1b* gene were used to standardize the expression levels of all genes in our quantitative RT‐PCR experiments. Therefore, the results of two experiments can be comparable. In our study, we observed that the expression levels of *Glyma.10g168900* and *Glyma.20g220800* genes containing Promoter 2 (P2) and Promoter 3 (P3), respectively, in roots around 10‐fold of the transcript levels of the *ELF1b* gene (Figure S10). Surprisingly, the transcript levels of the *P2‐GmDR1* and *P3‐GmDR1* transgenes were over 500‐fold higher than that of the *ELF1b* gene (Figure 2a; Figure S10). Thus, a 50‐fold increases in activities of the two promoters were observed among the transgenic lines carrying the two transgenes (Figure 2a). We evaluated 15 independent transformants for *P2‐GmDR1*, and eight for *P3‐GmDR1* transgene. From these transgenic lines, we identified the most SDS‐resistant transgenic lines for molecular analyses. Enhanced activities of Promoters 2 and 3, measured by *GmDR1* transcript level, among the transgenic lines might have evolved from the position effect variegation phenomenon (Berloco *et al*., 2014; Reuter and Spierer, 1992). The enhancer and silencer elements as well methylation activities of the T‐DNA insertion sites can influence the expression of transgenes. Position effect‐induced variegation is often observed in transgenic studies (Bhattacharyya *et al*., 1994; Wakimoto, 1998; Williams *et al*., 2008). Study of a large number of transgenic events allowed us to identify the desirable transgenic plants with enhanced SDS resistance resulting from very strong overexpression of *GmDR1*.

Overexpression of *GmDR1* however did not result any noticeable undesirable effect on the soybean plants. Both plant height and seed yield were not affected among the transgenic plants (Figure S4). Overexpression of *GmDR1* led to constitutive induction of a novel aphid resistance‐related novel gene *GmAI1* in addition to defence mechanisms including SA‐mediated defence pathway (Figure 2; Figure S13e; Studham and MacIntosh, 2013). A transcriptomic study of the transgenic lines is warranted to gain a comprehensive understanding of the defence pathways constitutively induced by overexpressed‐*GmDR1* among the transgenic lines.

We have demonstrated that GmDR1 is an integral plasma membrane protein (Figure 6). GmDR1 with 73 amino acid residues contains an N‐terminal cytoplasmic domain (11 aa), two predicted transmembrane domains (23 aa), an ecto‐domain (14 aa), and a short cytoplasmic tail (2 aa) (Figure 6a; Figure S14b). GmDR1 is a member of the Panther family PTHR33659:SF7 that contains twenty‐five uncharacterized genes including four from soybean (*GmDR1, −2, −3, −4*). Plasma membrane residence and priming of multiple defence mechanisms to enhance broad‐spectrum pathogen and pest resistance indicate that most likely GmDR1 is a receptor that recognizes pathogen and pest‐associated molecular pattern(s). One pathogen‐associated molecular pattern (PAMP) common to all four organisms, two pathogens and two pests, is chitin (Bos *et al*., 2010; Chen and Peng, 2019; Sánchez‐Vallet *et al*., 2015; Zhou *et al*., 2017). Chitin application to intact soybean plants significantly enhanced the accumulation transcripts of marker genes of the SA and JA‐regulated defence pathways, whereas suppressed the transcription of a marker gene of the defence pathway mediated by ethylene (Figure 7). We failed to see induction in any of the SA or JA marker genes in response to chitin treatment among the transgenic line carrying the *GUS* gene or nontransgenic Williams 82 line. Lack of endogenous *GmDR1* gene‐mediated induction of SA and JA markers among these lines could be attributed to very low expression levels of this receptor protein gene (Figure 7a). Because of overexpression, the *GmDR1* transgenes were able to show the chitin responses as well as broad‐spectrum disease and pest resistance among the transgenic lines (Figure 7a).

We, therefore, hypothesize that one of the genetic mechanisms involved in generating broad‐spectrum resistance among the transgenic soybean lines with overexpressed *GmDR1* transgenes could be through activation of defence pathways mediated by chitin, a well‐recognized PAMP (Wan *et al*., 2008b).

In our study, we infected roots with *F. virguliforme* and treated leaves with chitin. We observed variations in the expression patterns of genes including *GmDR1*, *GmEDS1b, GmJAR1* and *GmPR1s* following infection with *F. virguliforme* and treatment with chitin. These differences may be arisen because of variation in organ types studied and complexity of *F. virguliforme* infection as compared to the chitin treatment (Figures 2,7). Pathogenicity factors of *F. virguliforme* are involved in developing SDS and interfere the host defence mechanisms. These results may also indicate that *GmDR1*‐induced broad‐spectrum resistance is governed by multiple genetic mechanisms including the ones induced by chitin.

Transgenic studies showed that chitin induced defence responses are specific to constitutively overexpressed *GmDR1* and are absent in nontransgenic Williams 82. We, therefore, hypothesize that one of the mechanisms involved in generating broad‐spectrum resistance among the transgenic lines could be through activation of multiple defence pathways mediated by chitin, a well‐recognized PAMP, through a possible interaction with the overexpressed GmDR1 protein (Wan *et al*., 2008b). GmDR1, therefore, could be a PAMP recognition receptor. It does not seem to contain a typical LysM domain for binding chitin and peptidoglycans found in LysM‐containing receptor‐like kinases (Buist *et al*., 2008; Petutschnig et a., 2010; Tanaka *et al*., 2013). Further studies warranted to confirm the possible interaction of GmDR1 with chitin and establish its PRR role in generating broad‐spectrum disease and pest resistance in soybean.

Natural spider mite resistance has not yet been identified in most crop plants including soybean (http://corn.agronomy.wisc.edu/Management/pdfs/A3890.pdf; Agut *et al*., 2018). Biological and chemical controls are the major methods of managing this serous pest (Agut *et al*., 2018). The generated spider mite resistance through overexpression *GmDR1* is novel and has the potentiality in breeding spider mite‐resistant legumes, cotton, cocoa and jute, in which *GmDR1* homologues are detected (Figure S12). It will also be important to investigate the potentiality of *GmDR1* in creating novel spider mite resistance in other crop species that do not carry any *GmDR1* homologs.

## Experimental procedures

### Binary vector constructions and generation of soybean transgenic lines

*GmDR1* was fused to three promoters as follows. The pTF102 binary vector (Frame *et al*., 2002) was modified by replacing the CaMV 35S promoter with any one of the three selected promoters: promoter (prom) 1 (*Glyma18g47390*), prom 2 (*Glyma10g31210*) and prom 3 (*Glyma20g36300*) (Figure S1a; Table S1; Ngaki *et al*., 2016). Primers used for amplifying and cloning these new promoters are listed in Table S5. The *GUS* gene in the modified pTF102 vector was replaced with the genomic sequence of the *GmDR1* gene (Figure S1a). The created plasmids therefore contained the *GmDR1* fusion genes generated by fusing *GmDR1* to any of the above three promoters. The CaMV 35S polyA signal was fused at the 3’‐end of *GmDR1* for polyadenylation of the transgene transcripts. The GmDR1‐Fw and GmDR1‐Rev primers were used to amplify *GmDR1*. The created binary plasmid vectors were introduced into *Agrobacterium tumefaciens* strain EH101 through electroporation.

Seven independent transformants for *P1‐DS1*, 15 for *P2‐DS1* and eight for *P3‐DS1* transgenes were generated by conducting *Agrobacterium*‐mediated transformation of the Williams 82 soybean cultivar at the Plant Transformation Facility, Iowa State University (Paz *et al*., 2004). Transgenic R_0_ plants resistant to the glufosinate‐ammonium (Liberty 280 SL, Bayer CropScience, Research Triangle Park, NC, USA) were grown in individual pots containing the standard soil fertilized with Osmocote (Scotts, Marysville, OH, USA) in a greenhouse under 16 h light and 8 h dark photoperiod. To confirm the transgene insertion, genomic DNA was extracted from young leaves of R_0_ plants (Ngaki *et al*., 2016). PCR analysis was conducted to determine the integration of *GmDR1* and *bar* (bialaphos resistance) genes into the soybean genome (Figure S1b). The R_1_ seeds from individual R_0_ plants carrying the *GmDR1* and *bar* genes were harvested.

### *F. virguliforme* infection assay in growth chambers

*F. virguliforme* Mont‐1 was grown on 1/3 potato dextrose agar (PDA) plates for 3 weeks. The inoculum was prepared by growing the pathogen in sorghum meals (Luckew *et al.,* 2012). The inoculum was well mixed at a concentration of 1: 20: inoculum: sand and soil mixture in equal proportion and placed in 237‐ml Styrofoam cups. Three independent experiments were carried out in the growth chambers maintained at 22.5 ºC, 16 h light with 350 µE/m2/s intensity and 8 h dark photoperiod. The SDS susceptible Williams 82 and the SDS‐resistant MN1606 were grown along with the transgenic lines as controls. Plants were watered daily and SDS symptoms were scored as follows. Foliar and root rot symptoms were evaluated 4 weeks after planting according to the published methods (Hartman *et al.,* 1997; Huang and Hartman, 1998; Li *et al*., 2009). The plants showing a score of 1 (no symptoms) or 2 (slight yellowing) were considered SDS‐resistant plants, whereas plants with scores 3 to 7 (browning, interveinal chlorosis and necrosis) were classified as the susceptible plants (Table S6). Thirty‐seven days after planting (DAP), plants were carefully removed from the cups and the roots were washed in warm tap water. The root showing dark brown to black discoloration (Roy *et al.,* 1997) was visually assessed in a percentage scale from 0 to 100% of the total root area with an increment of 5%; 0% means healthy roots with light brown colour to 100% means rotten roots with dark black colour. Root tissues of resistant and susceptible progeny plants of each transgenic line were collected and immediately frozen in liquid nitrogen for molecular analysis.

### Field trials for responses of transgenic soybean lines to *F. virguliforme*

In the summer of 2015 (June 11 to October 30, 2015), seeds of the R_1_ progenies of independent transformants, Williams 82 and MN1606 were hand‐planted along with *F. virguliforme* inoculum to evaluate their responses to the pathogen in a completely randomized block design with two replications. The experiment was conducted at the Hinds Research Farm, Iowa State University located in north of Ames, Iowa. The inoculum of *F. virguliforme* isolate NE305S was prepared the same way as for Mont‐1 used in the growth chamber experiments. For each transgenic event, twenty‐five seeds were mixed with 10 ml dry *F. virguliforme* inoculum and planted using a push planter. To eliminate the segregants with no transgenes, transgenic plants were sprayed with the Liberty 280L solution (glufosinate‐ammonium at a final concentration of 250 mg/L and 0.1% Tween 20) 2 weeks following germination (Ngaki *et al*., 2016). The spray was repeated once more after two days of the first application. Twelve herbicide‐resistant plants were randomly selected from each transgenic event for collecting young leaves to prepare DNA for transgene copy number study as reported earlier (Ngaki *et al*., 2016). SDS symptoms appeared in August and were scored as described for growth chamber experiments; and plants were classified into resistant with scores 0 to 2 and susceptible with scores 3 to 7 (Table S6).

In the summer of 2016 (June 7 to October 30, 2016), we conducted the field trial for R_2_ transgenic plants. The method was similar to the 2015 field experiment, but this time we planted R_2_ seeds of selected putative homozygous R_1_ plants carrying a single transgene copy (Ngaki *et al*., 2016) in four blocks. We sprayed Liberty herbicide to eliminate any possible heterogeneous R_1_ families.

In the summer of 2017 (May 31 to October 31, 2017), the third field trial was conducted for the R_3_ generation of transgenic soybean plants. The methods followed for the field trial were similar to that of the field trials conducted in the previous years. All progenies of the homozygous R_3_ lines were found to be resistant to the Liberty herbicide.

For the summer 2018 (May 31 to October 31, 2018), we tested the R_4_ generation of transgenic soybean plants. We followed the same methods as in the previous years, except that the experiment was conducted at the Iowa State University Horticulture Research Station located on 55519 170^th^ St. in the north of Ames, IA 50010. As expected, all progenies of the homozygous R_4_ lines were found to be resistant to Liberty herbicide.

## Conflict of interest

The authors declare no competing financial interests.

## Author contributions

M.K.B. conceived and supervised the project. M.K.B. and M.N.N. designed the experiments. M.N.N. cloned the *GmDR1* gene and conducted all experiments related to the generation, confirmation and characterization of transgenic soybean plants, the aphid bioassay, the qPCR and qRT‐PCR analysis, the subcellular localization of GmDR1, and the identification of co‐expressed genes. M.N.N. carried out SDS and soybean aphid assays in the growth chamber. M.N.N. and B.W. conducted the field trials. M.N.N. and D.K.S. performed the mite inoculation assays. M.N.N, D.K.S, and B.W. conducted SCN inoculation assays. M.N.N. carried out the microscopic study of the SCN infection. B.W. generated the predicted protein structure and phylogenic tree. M.N.N. and M.K.B. analysed the data. M.N.N. and M.K.B. wrote the manuscript. All authors reviewed the manuscript.

## Supporting information


**Figure S1** Binary vector plasmids and PCR confirmation of transgenic soybean plants carrying the *GmDR1* transgenes.
**Figure S2** Overexpression of *GmDR1* transgenes enhances SDS resistance under growth chamber conditions.
**Figure S3** Transgenic lines carrying *GmDR1* transgenes showed enhanced foliar SDS resistance under field conditions.
**Figure S4** Transgenic lines carrying *GmDR1* transgenes exhibited similar plant height and seed size and seeds per plant as in non‐transgenic Williams 82.
**Figure S5** Expression of *GmDR1* conferred immunity to two‐spotted spider mites.
**Figure S6** Transgenic soybean lines carrying *GmDR1* transgenes expressed resistance to two‐spotted spider mites.
**Figure S7** Transgenic soybean lines carrying *GmDR1* transgenes expressed resistance to soybean aphids.
**Figure S8** Transgenic soybean lines overexpressing *GmDR1* showed enhanced SCN resistance.
**Figure S9** Responses of transgenic soybean lines overexpressing *GmDR1* to SCN.
**Figure S10** Expression levels of the genes containing *Promoter 2* and *Promoter 3*.
**Figure S11** Expression of *GmDR1* transgenes in leaves of transgenic soybean plants.
**Figure S12** Phylogenetic tree and alignment of the *GmDR1* and its closely related homo‐ and homeologues.
**Figure S13** *GmAI1* is constitutively up‐regulated in *GmDR1* transgenic lines.
**Figure S14** Putative structure of GmDR1.
**Figure S15** Sub‐cellular localization of GmDR1.
**Table S1** Description of the three promoters used in generating the *GmDR1* fusion genes.
**Table S2** Expression levels of three soybean genes in soybean roots following *F. virguliforme* infection.
**Table S3** *GmDR1* homo‐ and homeologoues.
**Table S4** *GmDR1*‐co‐expressed genes with Pearson correlation coefficient ≥ 0.9 (www.phytozome.jgi.doe.gov).
**Table S5** Primers used in this study.
**Table S6** SDS foliar disease severity scale.

## Data Availability

The authors declare that all the data supporting the findings of this study are available upon request. Additional information relating to (i) *F. virguliforme* infection assay in growth chambers; (ii) field trials for responses of transgenic soybean lines to *F. virguliforme*; (iii) two‐spotted spider mite (*Tetranychus urticae*) bioassay; (iv) abiotic treatments; (v) measurements of chlorophyll; (vi) DNA extraction and PCR analysis; (vii) RNA extraction and RT‐PCR analysis; (viii) quantitative PCR (qPCR); (ix) subcellular localization of the GmDR1 protein; and (x) bioinformatics and statistical analyses can be found in the Supplementary Experimental Procedures.
