# Supplementary material for: Overexpression of a plasma membrane protein generated broad‐spectrum immunity in soybean
Source: Plant Biotechnol J. 2020 Oct 9;19(3):502–16. doi: 10.1111/pbi.13479 (PMC7957895; doi:10.1111/pbi.13479)
Supplement: Supplementary file 1 — Figure S1 Binary vector plasmids and PCR confirmation of transgenic soybean plants carrying the GmDR1 transgenes. Figure S2 Overexpression of GmDR1 transgenes enhances SDS resistance under growth chamber conditions. Figure S3 Transgenic lines carrying GmDR1 transgenes showed enhanced foliar SDS resistance under field conditions. Figure S4 Transgenic lines carrying GmDR1 transgenes exhibited similar plant height and seed size and seeds per plant as in non‐transgenic Williams 82. Figure S5 Expression of GmDR1 conferred immunity to two‐spotted spider mites. Figure S6 Transgenic soybean lines carrying GmDR1 transgenes expressed resistance to two‐spotted spider mites. Figure S7 Transgenic soybean lines carrying GmDR1 transgenes expressed resistance to soybean aphids. Figure S8 Transgenic soybean lines overexpressing GmDR1 showed enhanced SCN resistance. Figure S9 Responses of transgenic soybean lines overexpressing GmDR1 to SCN. Figure S10 Expression levels of the genes containing Promoter 2 and Promoter 3. Figure S11 Expression of GmDR1 transgenes in leaves of transgenic soybean plants. Figure S12 Phylogenetic tree and alignment of the GmDR1 and its closely related homo‐ and homeologues. Figure S13 GmAI1 is constitutively up‐regulated in GmDR1 transgenic lines. Figure S14 Putative structure of GmDR1. Figure S15 Sub‐cellular localization of GmDR1. Table S1 Description of the three promoters used in generating the GmDR1 fusion genes. Table S2 Expression levels of three soybean genes in soybean roots following F. virguliforme infection. Table S3 GmDR1 homo‐ and homeologoues. Table S4 GmDR1‐co‐expressed genes with Pearson correlation coefficient ≥ 0.9 (www.phytozome.jgi.doe.gov). Table S5 Primers used in this study. Table S6 SDS foliar disease severity scale. [file PBI-19-502-s001.docx]

# Supporting Information

(Supplementary Experimental Procedures, Supplementary Figures 1 to 15, and Supplementary Tables 1 to 6)

Overexpression of a plasma membrane protein generated broad-spectrum immunity in soybean.

Micheline N. Ngaki^a^, Dipak K. Sahoo^a^, Bing Wang^a,b^ & Madan K. Bhattacharyya^a,1^

^a^Department of Agronomy, Iowa State University, Ames, Iowa, USA.

^b^Present address: Department of Energy, Joint Genome Institute, Walnut Creek, California, USA.

^1^To whom correspondence may be addressed. Email: mbhattac@iastate.edu.

Supplementary Experimental Procedures

**Soybean aphid (*Aphis glycines*) bioassay**

We obtained the soybean aphids Biotype 1 from the MacIntosh laboratory, Iowa State University and conducted aphid inoculation according to Morriss *et al.* (2017). Aphid inoculated plants were grown in pots, maintained in a growth chamber at 25 °C under 16 h light and 8 h dark photoperiod. To generate uniformly aged cohort of aphids, we infested the abaxial side of young Williams 82 leaves with 35 mature females protected by a clip-cage (product # 1458, BioQuip Products, Rancho Dominguez, CA, USA) (Figure S7a). On day 1, the mothers were removed and neonates kept on the caged leaf for 5 days. The growth of these aphids was monitored daily. On day 5, the caged aphids were used to infest experimental plants including transgenic soybean lines carrying the *GmDR1* transgenes, the aphid susceptible cultivar Williams 82, and the aphid resistant LD05-16060 line carrying the *Rag1* gene (Wiarda *et al.,* 2012; Bansal *et al.,* 2014). We placed 10 soybean aphids on the central part of each leaflet of the second trifoliate of three to four-week-old plants and restricted them using the clip cages for 7 days. The number of aphids and nymphs on each infected leaf was then counted under an Olympus stereomicroscope. At least six R_1_ plants for each independent *GmDR1* transgenic R_0_ plant were used in each of the four clip-cage experiments. Inoculated plants were completely randomized in a growth chamber.

**SCN (*Heterodera glycines*) bioassay**

Transgenic lines were evaluated for possible SCN resistance according to a method developed in Tylka Laboratory, Iowa State University. Soil collected from Muscatine, Muscatin County, Iowa, containing ~50 cysts of the SCN HG type 2.5.7 (Race 5) was used to fill up cone-tainers (Hummer international, St. Joseph, MO, UDA) (Tylka, 2016; Niblack *et al.,* 2002). One R_1_ or R_2_ seed was sown in each Muscatine soil filled cone-tainer. For each transgenic line, 16 to 30 seeds were planted in the SCN infected soil. The cultivar Williams 82 and IA2050 (highly SCN susceptible) and A95-684043 (highly SCN resistant) (Donald *et al.,* 2006) were used as controls. Cone-tainers were randomly distributed in a water bath set at 27 ºC under natural light conditions. Plants were watered daily and cysts were harvested at the end of the SCN life cycle; i.e., thirty days following planting. Cysts were gently washed from roots with high-pressure tap water and collected on nested sieves of 20 mesh (850 µm pore) placed over sieves of 60 mesh (250 µm pore), and then transferred to a plastic beaker. Cysts were counted under a stereoscopic microscope NI-150 (Nikon Instruments Inc.). The female indices (FI) were calculated as follows: (number of cysts per plant X 100) / (mean number of cysts on the susceptible control, Williams 82) (Schmitt and Shannon, 1992; Kim *et al.,* 2010). Transgenic lines were then rated as highly resistant (R) with FI ≤ 10, moderately resistant (MR) with FI ranging between 11 and 29, moderately susceptible (MS) with FI between 30 and 60, and susceptible (S) with FI > 60 (Guo *et al*., 2015).

SCN infection process was monitored at 10, 12, and 15 days following inoculation of homozygous R_3_ progenies, Williams 82 and A95-684043. Roots of six SCN infected-plants of each homozygous R_3_ plant along with that of control plants were harvested at each of the three developmental stages. Nematodes of the infected roots were stained using a published method (Bybd *et al.,* 1983). Root tissues were cleared in chlorine bleach (NaOCl), then nematodes were stained with fuchsine acid, and finally roots were distained by boiling in acidified glycerin. Stained nematodes were then counted under an Olympus stereomicroscope.

**Two-spotted spider mite (*Tetranychus urticae*) bioassay**

The mite colony, obtained naturally in greenhouse, was reared on susceptible Williams 82 plants grown in the ISU Agronomy Department greenhouse for inoculation of soybean plants. Experimental plants were grown in plastic pots in a spider-mite free growth chamber using standard soil.

For mite-inoculation experiments, two or three-week old R_1_ soybean transgenic plants and Williams 82 plants were infested using a modified published mite assay (Škaloudová *et al.,* 2006). One hundred adult female spider mites were identified from the mite culture using a magnifying glass and transferred with a fine painting brush to the third trifoliate. Symptoms were evaluated two weeks and two months following infestation.

For leaf disc assay, homozygous R_2_ transgenic and Williams 82 plants were grown for four weeks in a spider mite-free growth chamber. The third trifoliate of each plant was used for the mite bioassay (Miyazaki *et al.,* 2014). Three leaf discs (one from each leaflet) were generated from each trifoliate and inoculated with: (i) 0 (control), (ii) ten, or (iii) 40 adult female mites on the abaxial side of the leaf discs, placed on wet filter papers in Petri dishes. Following inoculation, Petri dishes were sealed and maintained in a growth chamber under 25 °C and, 18 h light and 6 h dark photoperiod for five days. Spider mite eggs on the leaf discs were counted under an Olympus stereomicroscope.

**Abiotic treatments**

Chitin azure was purchased from Sigma-Aldrich. We fed stem-cut two-week-old soybean seedlings with 100 µmol/L of chitin solution in phosphate buffer (15 mmol/L sodium phosphate, pH 6.5) (Orozco-Cardenas and Ryan, 1999; Khan *et al*., 2003). Stems of the seedlings were cut just above ground with a razor blade and fed with 0.5 mL chitin solution in Eppendorf tubes. As soon as the solution was completely taken-up by the cut seedlings, the seedlings were transferred to 20 mL of distilled water in 50 ml plastic tube; and leaf samples were collected 12 and 24 h after chitin treatment and immediately placed in liquid nitrogen for RNA extraction. In controls, only phosphate buffer was fed to the cut soybean seedlings.

**Measurements of chlorophyll**

We quantified the chlorophyll content in the mite-infested leaves of resistant transgenic and susceptible non-transgenic Williams 82 plants. Leaf discs of approximately 1 cm^2^ were excised, placed in Eppendorf tubes, and frozen overnight at –80°C. Then, 1 ml of 80% acetone was added to each tube and incubated at room temperature in darkness for 5 days. The absorbency of the acetone solution was measured at 645 and 663 nm. The amount of chlorophyll was calculated according to a published method (Arnon, 1945).

**DNA extraction and PCR analysis**

PCR analysis was conducted on genomic DNA extracted from young leaves of soybean plants including R_0_, R_1_, and R_2_ plants using a CTAB protocol (Ngaki *et al.,* 2012). Primer sequences are listed in Table S5. The PCR conditions for genomic DNA included an initial cycle of 94 °C for 2 min, and then 35 cycles of 94 ⁰C for 30 sec, 55 ⁰C for 30 sec, and 72 ⁰C for 1 min, and a final step of 72 ⁰C for 10 min. The PCR products were examined by agarose gel electrophoresis.

**RNA extraction and RT-PCR analysis**

Total RNA samples were isolated from soybean leaf and root tissues using the SV total RNA Isolation System (Promega, Inc., Madison, WI, USA) that includes a DNase I treatment. cDNAs were synthesized using the SuperScript® III Reverse Transcriptase (ThermoFisher Scientific, Waltham, MA, USA). A soybean *ELF1b* (elongation factor 1β, *Glyma02g44460*) gene was used as an endogenous control. Primers used for all RT-PCR and PCR amplifications are presented in Table S5. We used gene-specific primers (Table S5) to distinguish the *GmDR1* transgenes from the endogenous *GmDR1* gene. For *GmDR1* transgenes, the forward primer was specific to the exon and the reverse primer to the CaMV 3’-end of pTF102. For the endogenous gene, the forward primer was specific to the 3’ UTR sequence, absent in the transgenes. In some cases, expression levels of genes were quantified by analyzing the scanned RT-PCR-gels with the ImageJ program (<http://imagej.nih.gov/ij/>) (Abramoff, 2004).

**Quantitative PCR (q- PCR)**

To determine the levels of *F. virguliforme* infection, roots of transgenic soybean plants carrying *GmDR1* transgenes and non-transgenic Williams 82 were harvested 15 d following planting of seeds in soil containing *F. virguliforme* (Mont-1 isolates) inoculum or no inoculum in a growth chamber. Fungal biomass in infected root tissues was quantified by conducting a genomic DNA-qPCR as follows. DNA was diluted to 20 ng/µl for qPCR in an iQ5 Biorad instrument using SYBR green. The single copy *F. virguliforme* *FvTox1* gene (Brar *et al.,* 2011; Mbofung *et al.,* 2011) was quantified as a measure of fungal biomass. To calculate the fold change of the *FvTox1* gene amplification, qPCR data of infected and corresponding uninfected roots were analyzed using the double delta threshold cycle (C_T_) method (Livak and Schmittgen, 2001) with the single copy soybean gene *Glyma.05G014200* (Ngaki *et al.,* 2016) as the internal control.

**Quantitative Real-time PCR (qRT-PCR)**

To examine the expression of *GmDR1* transgenes and defense-related genes among the transgenic plants, we carried out qRT-PCR on *F. virguliforme*-infected soybean roots and leaves treated with or without chitin. Total RNAs were extracted and converted into cDNAs as described above and diluted to a 1:9 ratio in sterile water. qRT-PCR analysis was conducted in an iQ5 Biorad instrument using SYBR green; and the double delta threshold cycle (C_T_) method (Livak and Schmittgen, 2001) was used with the *ELF1b* as the internal control to calculate the relative expression levels of individual genes.

**Delta delta CT (∆∆CT) method**

Each experiment was conducted three times. Three biological replications were included for each genotype and each biological replication of a genotype was run in triplicate. The delta delta C_T_ was calculated by using Microsoft excel according to Livak and Schmittgen (2001). At first, we calculated the average C_T_ values for each replication for the housekeeping gene and for the gene of interest for both the control (Willims 82) and the transgenic lines. Then we calculated the delta C_T_ (∆C_T_) value of a sample, which is the difference between the average C_T_ values of the gene of interest and the housekeeping gene of that sample. Next, we calculate the delta delta C_T_ (∆∆C_T_) value, which is the difference between a delta C_T_ of a treatment sample to the average delta C_T_ of the control (Williams 82). Finally, we used the formula “fold gene expression = 2^-(∆∆C_T_)” and average for each genotype was calculated.

**Subcellular localization of the GmDR1 protein**

To determine the subcellular location of the GmDR1 protein, the coding sequence of *GmDR1* was tagged to GFP at either the N- or C-terminus in the pISUAgron5 binary vector (modified pTF101 vector; Paz *et al.,* 2004) under the regulation of the CaMV 35S promoter. The coding sequence of *GmDR1* was amplified using GmDR1-GFP-Fw and GmDR1-GFP-Rev primers (Table S5). The created pISUAgron5 carrying the *GmDR1-GFP* or *GFP-GmDR1* fusion gene was transformed into the *A. tumefaciens* EH101 strain by electroporation for transient expression in Williams 82 and *Nicotiana benthamiana*. MCH (red fluorescence protein mCherry) tagged plasma membrane (PM) protein AtPIP2A (*AtPIP2A-MCH*) obtained from ABRC (Nelson *et al.,* 2007) was used as the plasma membrane (PM) marker.

Transient expression of the (i) *GmDR1-GFP* or *GFP-GmDR1* and (ii) *AtPIP2A-MCH* was conducted in young leaves of Williams 82 and *N. benthamiana* plants following syringe-infiltration of the *A. tumefaciens* EH101 strain carrying the individual fusion genes (Shamloul *et al.,* 2014; King *et al.*, 2015). Two days following *Agro*-infiltration and treatment with a drop of either (i) water or (ii) 5M NaCl for 5 minutes, the leaves were observed under a a Leica SP5 X MP confocal/multiphoton microscope (Leica Microsystems Inc., Buffalo Grove, IL USA) available at the Microscopy and NanoImaging Facility, Iowa State University. The GFP signals were detected at wavelengths 488 nm FITC and 568 nm TRITC. The mCherry signals were captured at wavelengths 587 to 610 nm (Schweiger and Schwenkert, 2014). The images were imported and processed using the MetaMorph image analysis software.

**Bioinformatics and statistical analyses**

*GmDR1* homologs were identified from the NCBI database using the BLASTX program. SignalP 4.1 server was used to predict the signal peptide. TMHMM Server v. 2.0 was used to predict the possible transmembrane domains and Protein Prowler 1.2, NCBI, NetPhos 2.0 server and Predotar v1.03 were used to analyze the sequences of GmDR1. Conserved protein domains were identified using Pfam and SMART programs.

For construction of the phylogenic tree, protein sequences were aligned using MUSCLE program. The phylogenetic tree was built as the maximum-likelihood tree using MEGA7 software. The 50% identity to *GmDR1* was used as the criterion in identifying the *GmDR1* homolog sequences. The evolutionary history was inferred using the Neighbor-Joining method (Saitou and Nei, 1987). The bootstrap consensus tree, inferred from 1,000 replicates (Zuckerkandl and Pauling, 1965), was used to represent the evolutionary history of the taxa analyzed (Zuckerkandl and Pauling, 1965). Branches corresponding to partitions reproduced in less than 50% bootstrap replicates are collapsed. The percentage of replicate trees in which the associated taxa clustered together in the bootstrap test (1,000 replicates) are shown next to the branches (Zuckerkandl and Pauling, 1965). The evolutionary distances were computed using the Poisson correction method (Kumar *et al*., 2016). All positions containing gaps and missing data were eliminated. There were a total of 50 positions in the final dataset. Evolutionary analyses were conducted in MEGA7 (Felsenstein, 1985).

For all experiments, statistical significance between transgenic and control lines was determined with the two-sample t-tests using R software (version 3.1.0).

Supplementary References

Abramoff M, Magelhaes P, Ram S (2004) Image processing with ImageJ. Biophotonics International 11:36–42.

Arnon DI (1945) Copper enzymes in isolated chloroplasts polyphenoloxidase in *Beta vulgaris*. *Plant Physiol* 20:1–15.

Bansal, R., Mian, M., Mittapalli, O. and Michel, A.P. (2014) RNA‐Seq reveals a xenobiotic stress response in the soybean aphid, Aphis glycines, when fed aphid‐resistant soybean. BMC Genom. 15, 972.

Brar HK, Swaminathan S, Bhattacharyya MK (2011) The *Fusarium virguliforme* toxin FvTox1 causes foliar sudden death syndrome-like symptoms in soybean. *Mol Plant Microbe Interact* 24:1179–88.

Bybd, D.W., Kirkpatrick, T., Barker, K.R. and Barker, K.R. (1983) An improved technique for clearing and staining plant tissues for detection of nematodes. J. Nematol. 15, 142–143.

Donald, P.A., Pierson, P.E., St.Martin, S.K., Sellers, P.R., Noel, G.R., MacGuidwin, A.E. et al. (2006) Assessing Heterodera glycines‐resistant and susceptible cultivar yield response. J. Nematol. 38, 76–82.

Felsenstein J (1985) Confidence limits on phylogenies: An approach using the bootstrap. *Evolution* 39:783-791.

Hartman G, Huang Y, Nelson R, Noel G (1997) Germplasm evaluation of *Glycine max* for resistance to *Fusarium solani*, the causal organism of sudden death syndrome. *Plant Dis* 81:515–518.

Huang YH, Hartman GL (1998) Reaction of selected soybean genotypes to isolates of *Fusarium solani* f. sp. *glycines* and their culture filtrates. *Plant Dis* 82:999–1002.

Khan W, Prithiviraj B, Smith DL (2003) Chitosan and chitin oligomers increase phenylalanine ammonia-lyase and tyrosine ammonia-lyase activities in soybean leaves. *J Plant Physiol* 160:859–863.

Kim, M., Hyten, D.L., Bent, A.F. and Diers, B.W..(2010) Fine mapping of the SCN resistance locus rhg1‐b from PI 88788. Plant Genome. 3, 81–89.

King JL, Finer JJ, McHale LK (2015) Development and optimization of Agroinﬁltration for soybean. *Plant Cell Rep* 34:133–140.

Kumar S, Stecher G, Tamura K (2016) MEGA7: Molecular evolutionary genetics analysis version 7.0 f or bigger datasets. *Mol Biol Evol* 33:1870-1874.

Li S, Hartman GL, Chen Y (2009) Evaluation of aggressiveness of *Fusarium virguliforme* isolates that cause soybean sudden death syndrome. *J Plant Pathol* 91:77–86.

Livak KJ, Schmittgen TD (2001) Analysis of relative gene expression data using real-time quantitative PCR and the 2–∆∆CT method. *Methods* 25:402–408.

Luckew AS, Cianzio SR, Leandro LF (2012) Screening method for distinguishing soybean resistance to *Fusarium virguliforme* in resistant X resistant crosses. *Crop Sci* 52:2215–2223.

Mbofung GCY, Fessehaie A, Bhattacharyya MK, Leandro, LFS (2011) A new TaqMan real-time polymerase chain reaction assay for quantification of Fusarium virguliforme in soil. *Plant Dis 95*:1420–1426.

Miyazaki J, Stiller WN, Truong TT, Xu Q, Hocart CH, Wilson LJ, Wilson IW (2014) Jasmonic acid is associated with resistance to two spotted spider mites in diploid cotton (*Gossypium arboreum*). *Funct Plant Biol* 41:748–757.

Morriss, S.C., Studham, M.E., Tylka, G.L. and MacIntosh, G.C. (2017) Validation of a hairy roots system to study soybean‐soybean aphid interactions. PLoS One, 12, e0174914.

Nelson BK, Cai X, Nebenführ A (2007) A multicolored set of in vivo organelle markers for co-localization studies in Arabidopsis and other plants. *Plant J* 51:1126–1136.

Ngaki MN, Louie GV, Philippe RN, Manning G, Pojer F, Bowman ME, *et al.* (2012) Evolution of the chalcone-isomerase fold from fatty-acid binding to stereospecific catalysis. *Nature* 485:530–533.

Ngaki MN, Wang B, Sahu BB, Srivastava SK, Farooqi MS, Kambakam S, *et al.* (2016) Transcriptomic study of the soybean-*Fusarium virguliforme* interaction revealed a novel ankyrin-repeat containing defense gene, expression of whose during infection led to enhanced resistance to the fungal pathogen in transgenic soybean plants. *PLoS One* 11:e0163106.

Niblack, T.L., Arelli, P.R., Noel, G.R., Opperman, C.H., Orf, J.H., Schmitt, D.P. et al. (2002) A revised classification scheme for genetically diverse populations of Heterodera glycines. J. Nematol. 34, 279–288.

Orozco-Cardenas M, Ryan CA (1999) Hydrogen peroxide is generated systemically in plant leaves by wounding and systemin via the octadecanoid pathway. *Proc Natl Acad Sci USA* 96:6553–6557.

Paz MM, et al. (2004) Assessment of conditions affecting *Agrobacterium*-mediated soybean transformation using the cotyledonary node explant. *Euphytica* 136:167–179.

Roy KW, Rupe JC, Hershman DE, Abney TS (1997) Sudden death syndrome of soybean. *Plant Dis* 81:1100–1111.

Saitou N and Nei M (1987) The neighbor-joining method: A new method for reconstructing phylogenetic trees. *Mol Biol Evol* 4:406–425.

Schmitt, D.P. and Shannon, G..(1992) Differentiating soybean responses to Heterodera glycines races. Crop. Sci. 32, 275–277.

Schweiger R, and Schwenkert S (2014) Protein-protein interactions visualized by bimolecular fluorescence complementation in tobacco protoplasts and leaves. *J Vis Exp* 85:51327.

Shamloul M, Trusa J, Mett V, Yusibov V (2014) Optimization and utilization of *Agrobacterium*-mediated transient protein production in Nicotiana. *J Vis Exp* 86:e51204.

Škaloudová B, Křivan V, Zemek, R (2006) Computer-assisted estimation of leaf damage caused by spider mites. *Comput Electron Agric* 53:81–91.

Tylka, G.L. (2016) A hypothetical human equivalent to the hg type. Plant Health Progr. 17, 149–151.

Zuckerkandl E and Pauling L (1965) Evolutionary divergence and convergence in proteins. Edited in *Evolving Genes and Proteins* by V. Bryson and H.J. Vogel, pp. 97-166. Academic Press, New York.


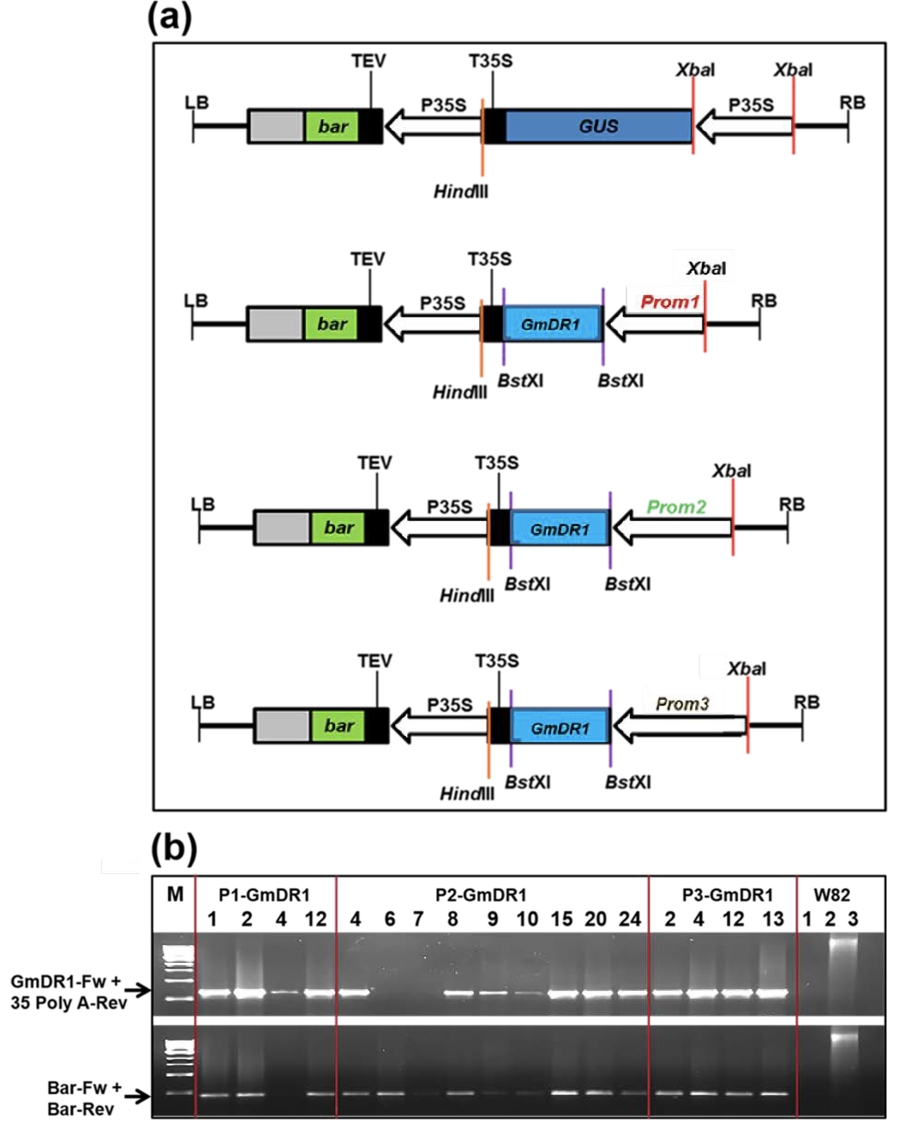


**Figure S1** Binary vector plasmids and PCR confirmation of transgenic soybean plants carrying the *GmDR1* transgenes. (a) pTF102-GmDR1 vector constructed for overexpression of *GmDR1* regulated by either Prom1 (P1), promoter 1, Prom2 (P2), promoter 2, or Prom3 (P3), promoter 3. T35S, CaMV 35S Poly A signal, was used at the 3’-end of the *GmDR1* fusion genes in the pTF102 binary vector (b) Genomic PCR confirming the insertion of *GmDR1* transgene and *bar* gene (bialaphos resistance gene). Gene-specific primers were as the forward primers and T35S 3’end-specific primer was used as the reverse primer for the PCR. M: DNA marker. The number under each transgene (e.g., *P1-GmDR1*) indicates the individual transformants (R_0_) (e.g. 1, *P1-GmDR1-1, 2, P1-GmDR1-2*, etc.)*.* W82, transgene receipient line Williams 82.

**
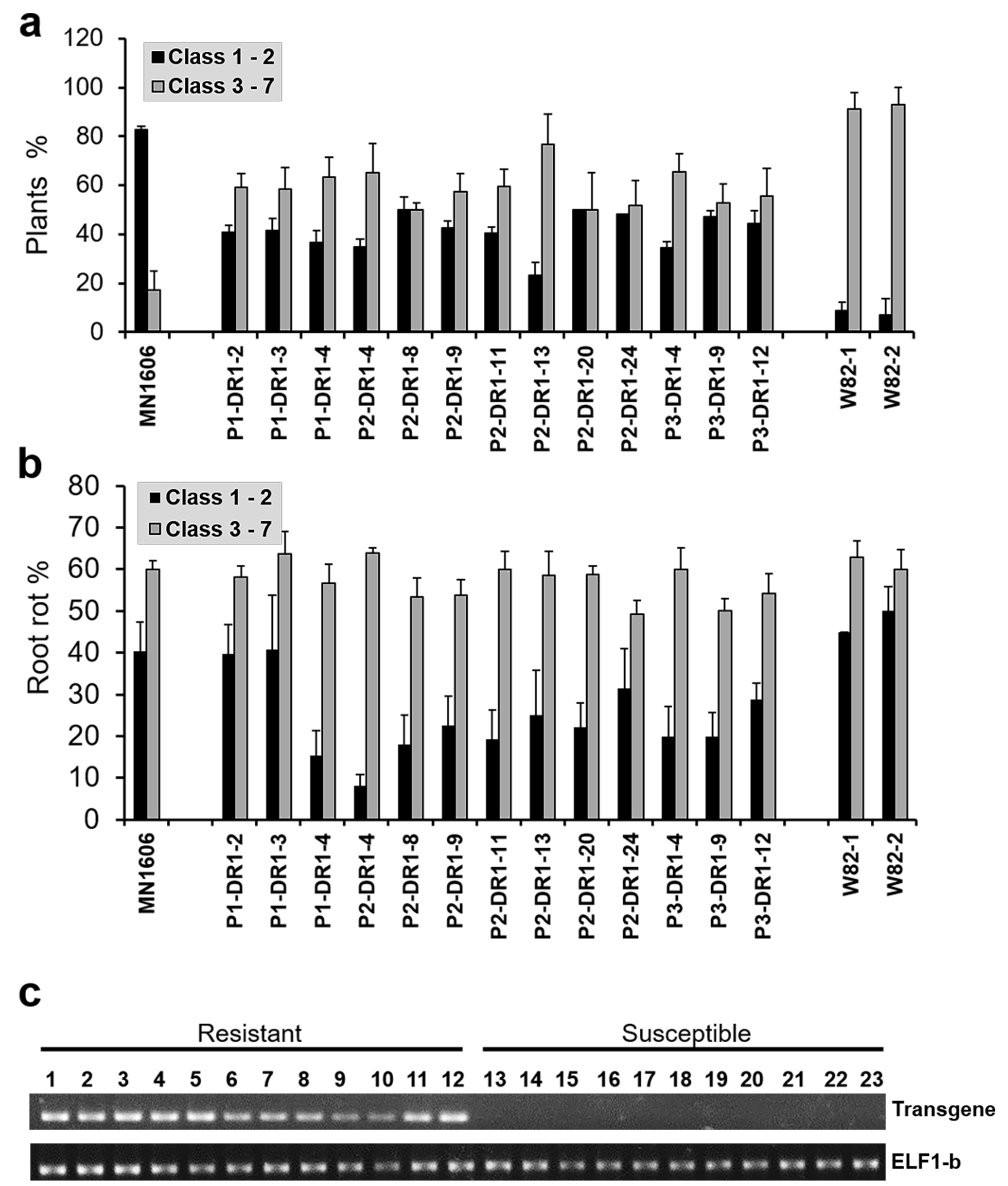
**

**Figure S2** Overexpression of *GmDR1* transgenes enhances SDS resistance under growth chamber conditions. (a) Foliar SDS of R_1_ plants scored four weeks following *F. virguliforme* inoculation. Approximately 25 to 60% (Class 1-2) of R_1_ transgenic plants showed enhanced foliar SDS resistance. Disease scores: 1, no symptoms; to 2, slight yellowing. The rest, Classes 3-7, showed severe disease symptoms with Score 3 for interveinal chlorosis to Score 7 for severe chlorosis to necrosis. Plants exhibiting scores 1 and 2 are considered resistant. Plants with scores 3 to 7 are susceptible. The recipient non-transgenic Williams 82 plant showed only 9% resistant plants. (b) Enhanced root resistance of transgenic plants to *F. virguliforme.* Root resistance to the pathogen is characterized by reduced blackening in the root-pith. Data in (a) and (b) are means of three independent experiments with n ≥ 15 R_1_ progenies per line in each experiment. (c) Expression of the *GmDR1* transgenes, detected by RT-PCR, among the SDS resistant and susceptible progenies randomly selected from individual transgenic plants. *F. virguliforme* infected roots of SDS resistant and susceptible plants were used in quantitative PCR analysis. (a). *ELF1b* (elongation factor 1β, *Glyma02g44460*) was used as the internal control. P1, P2, and P3 are promoter 1, promoter 2, and promoter 3. MN1606 is an SDS resistant line. Numbers 1 to 12 are selected SDS resistant plants, four for each transgene; 13 to 21 are selected SDS susceptible plants, three for each transgene; 22 and 23 are Williams 82 susceptible plants.

**
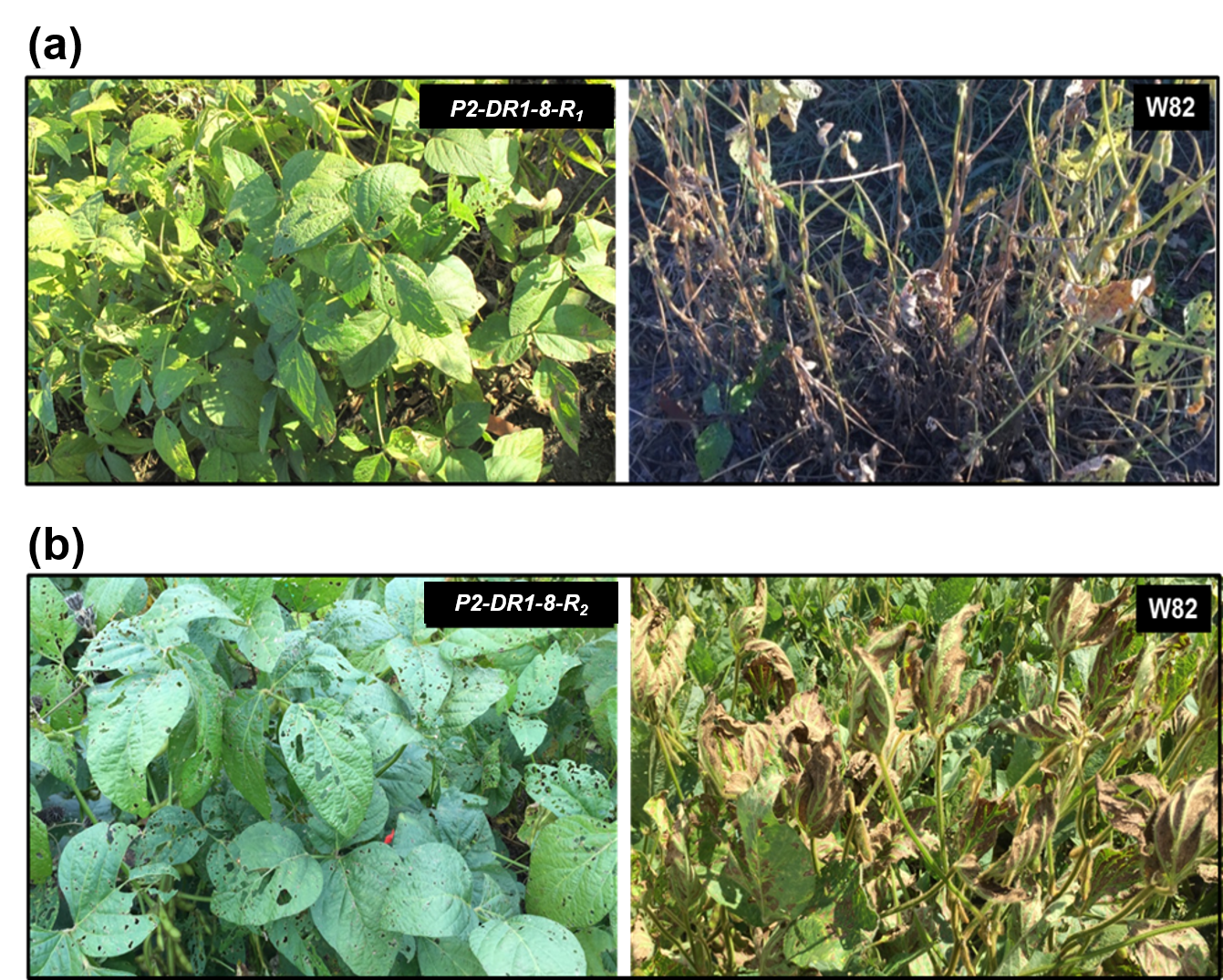
**

**Figure S3** Transgenic lines carrying *GmDR1* transgenes showed enhanced foliar SDS resistance under field conditions. (a) The *F. virguliforme* resistant *P2-DR1-R_1_* and susceptible Williams 82 (W82) lines in Hinds Farm, Ames, IA, in 2015. (b) *F. virguliforme* resistant *P2-DR1-R_2_* and susceptible control Williams 82 (W82) lines in Hinds Farm, in 2016.

**
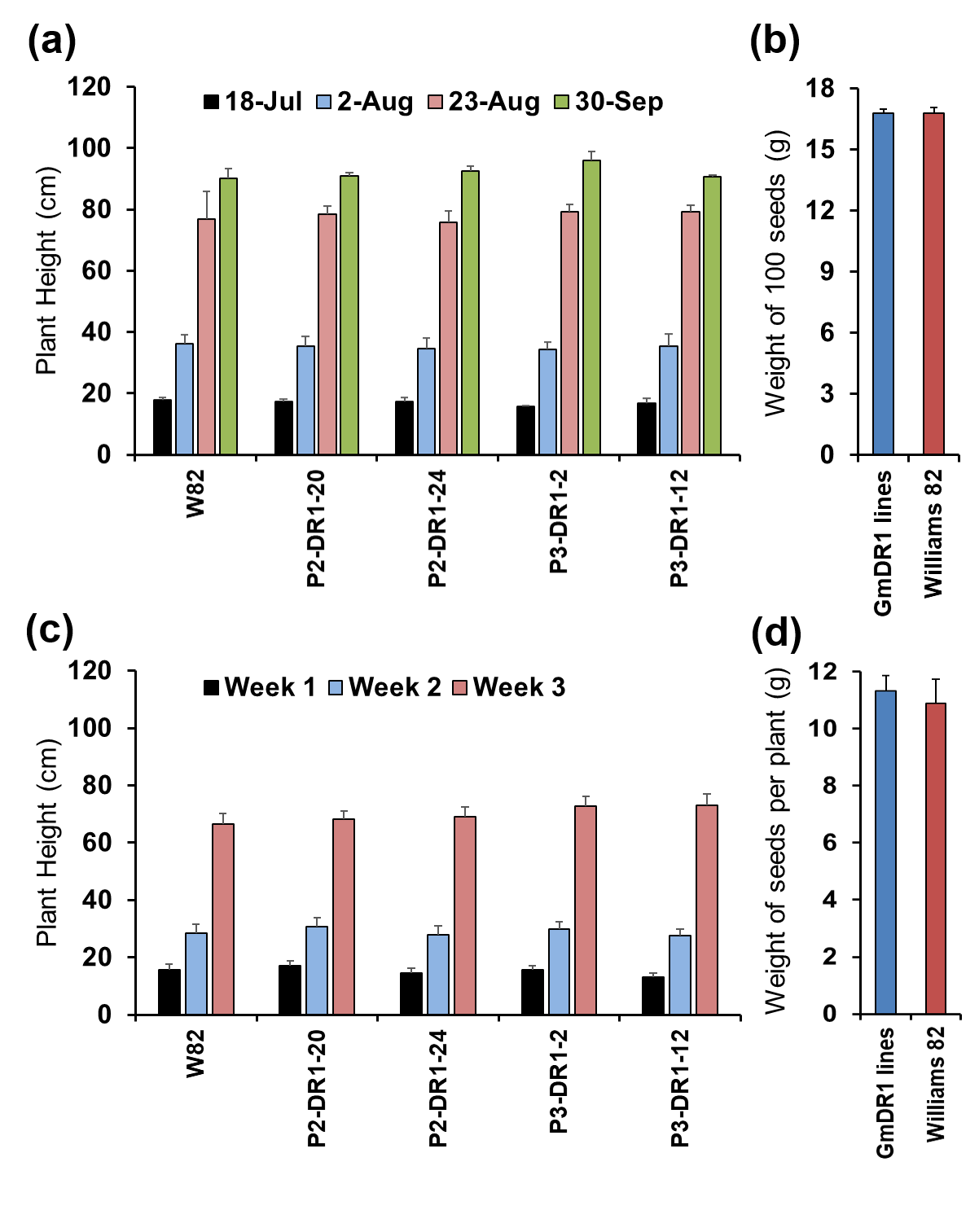
**

**Figure S4** Transgenic lines carrying *GmDR1* transgenes exhibited similar plant height and seed size and seeds per plant as in non-transgenic Williams 82. (a) Height of the plants in the field conditions. (b) Average weight of 100 seeds of all transgenic (*GmDR1* lines) and Williams 82 lines grown in the field. (c) Height of the plants grown in the greenhouse. (d) Average weight of seeds per plant for all transgenic (*GmDR1* lines) and Williams 82 lines grown in the greenhouse.


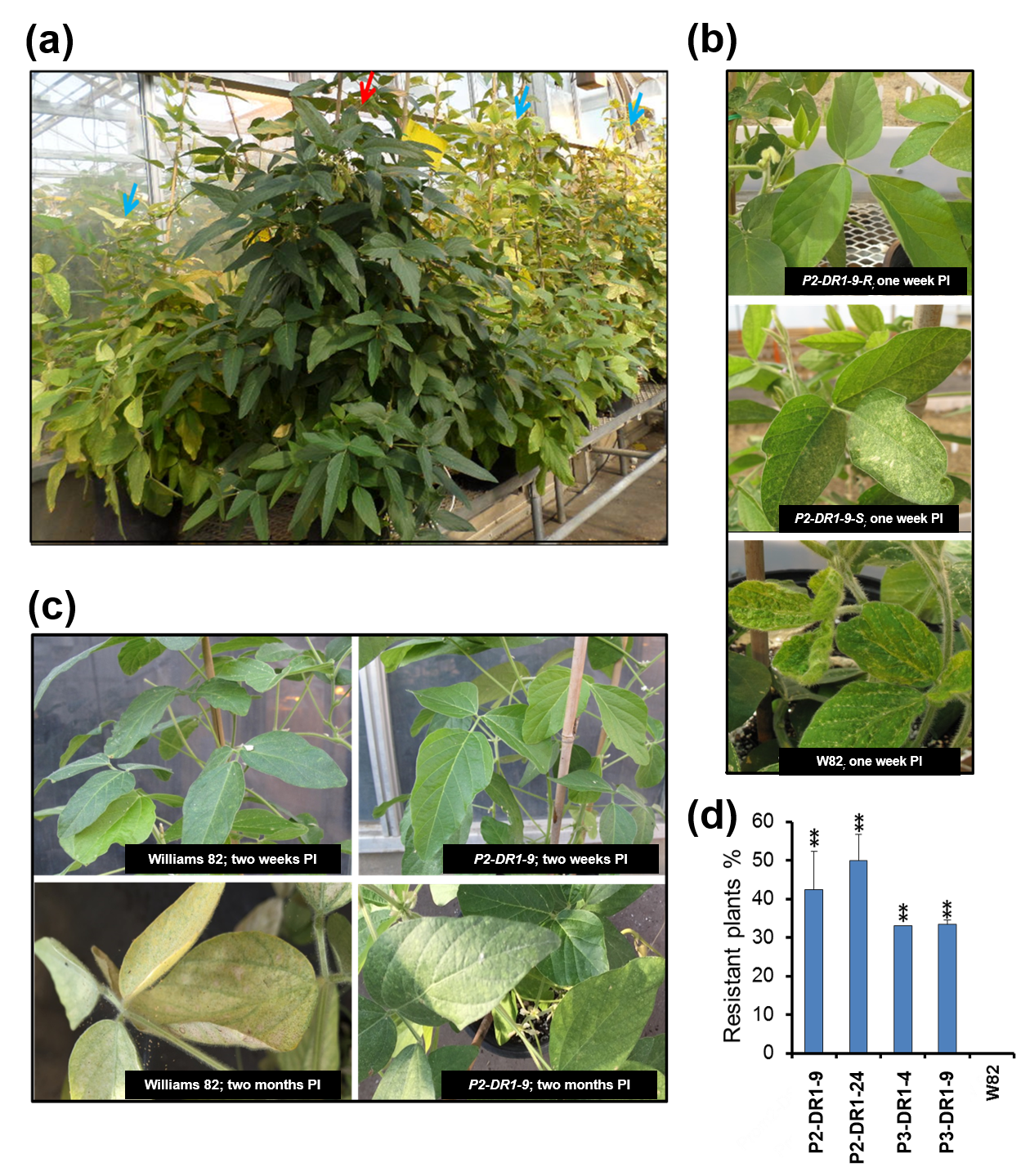


**Figure S5** Expression of *GmDR1* conferrs immunity to two-spotted spider mites. (a) An R_0_ transgenic soybean plant (red arrow) carrying *P2-DR1* transgene showed resistance to spider mite infestation. (b) Segregation of R_1_ progenies from a transformant one week following mite inoculation of two week-old plants (c) R­_1_ transgenic plants and Williams 82 two weeks and two months following spider mite inoculation. Leaves of three week-old plants were inoculated. (d) Percentage of mite resistant R_1_ progenies. *, significantly different from W82, Williams 82 at *p* ≤ 01. Data are mean and ± SE of two independent experiments. Ten R_1_ progenies of each transformant were evaluated. P2, and P3 are promoter 2, and promoter 3, respectively (Table S1).


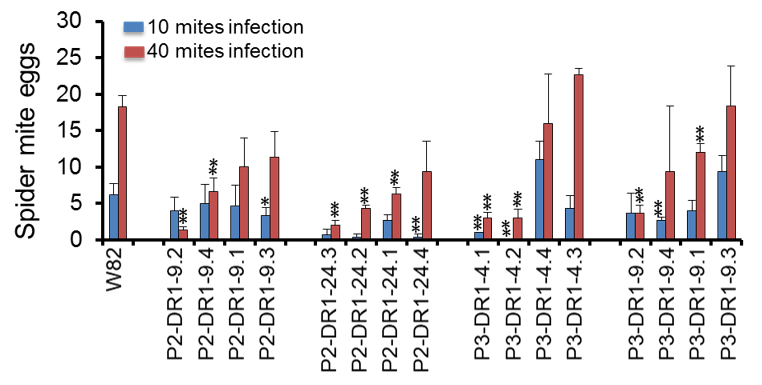


**Figure S6** Transgenic soybean lines carrying *GmDR1* transgenes expressed resistance to two-spotted spider mites. Number of two-spotted spider mite eggs on leaves of individual R_1_ segregating progenies five days following inoculation with either 10 or 40 adult mites. W82, Williams 82. No natural mite resistant soybean lines are available.

**
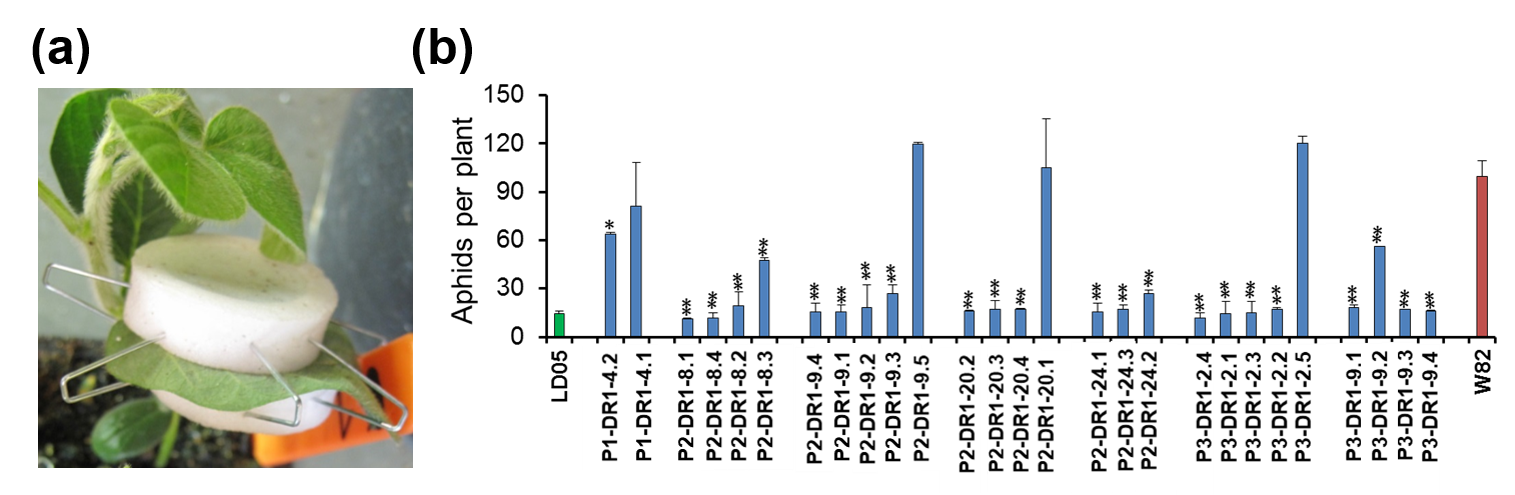
**

**Figure S7** Transgenic soybean lines carrying *GmDR1* transgenes expressed resistance to soybean aphids. (a) A clip cage on soybean leaf surface used for inoculation with soybean aphids. (b) Numbers of aphids on leaves of individual R_1_ segregating progenies seven days following inoculation with 10 soybean aphids, restricted to individual leaf blades using clip cages. LD05, LD05-16060 is a soybean aphid resistant soybean line. W82, Williams 82.


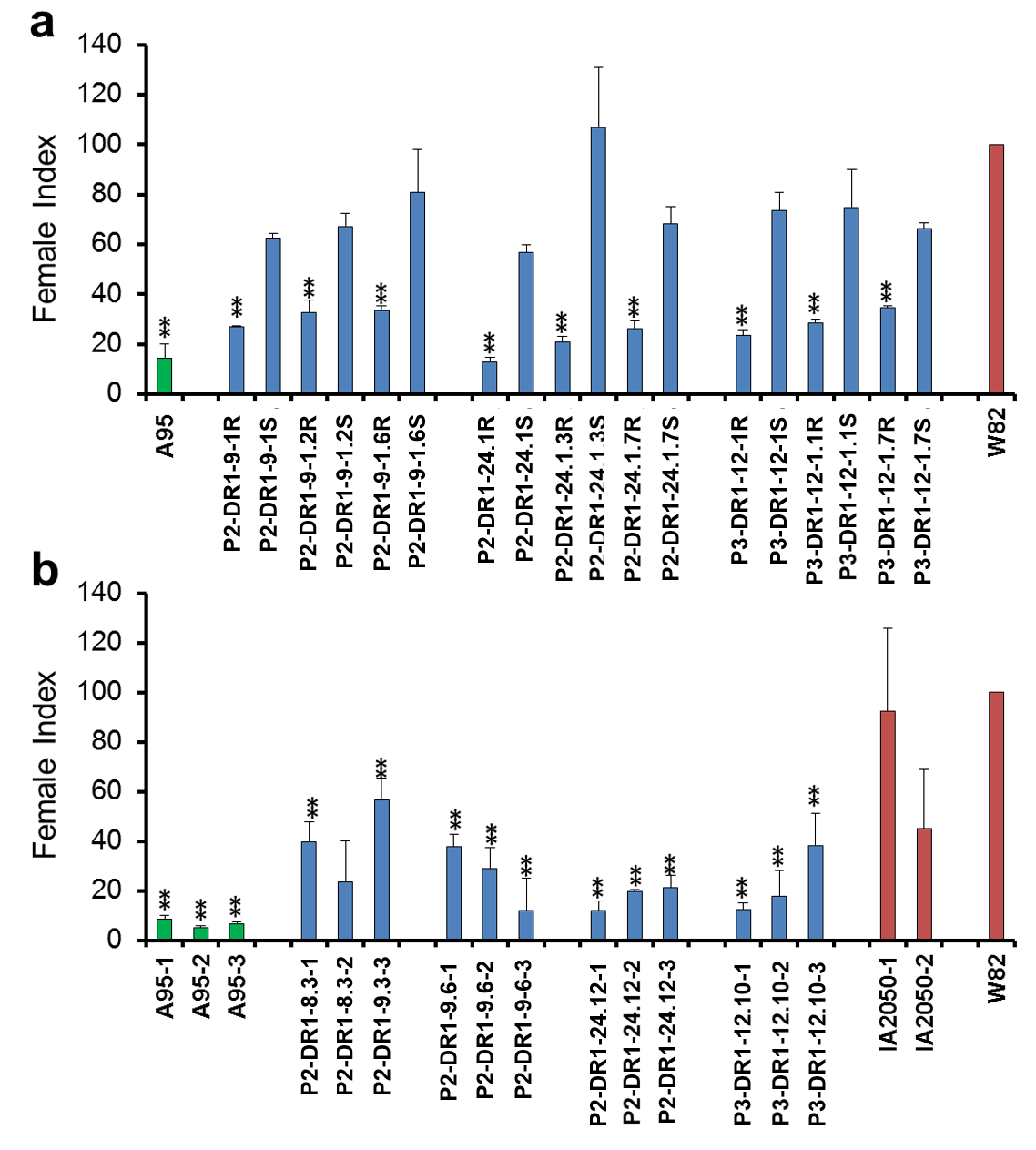


**Figure S8** Transgenic soybean lines overexpressing *GmDR1* showed enhanced SCN resistance. (a) Female indices of individual R_1_ segregating progenies. P2, and P3 represent promoter 2 and promoter 3, respectively. Data are means and ± SEs of two independent experiments, each with at least 15 plants. Transgenic lines are shown with blue bars, the resistant check A95, A95-684043, with a green bar, and the susceptible controls, W82 (Williams 82) and IA2050, are shown with red bars. (b) Female indices of individual R_2_ progenies. Data are averages and ± SEs of three independent experiments, each with six plants per line. *, statistically different at *p* < 0.01.


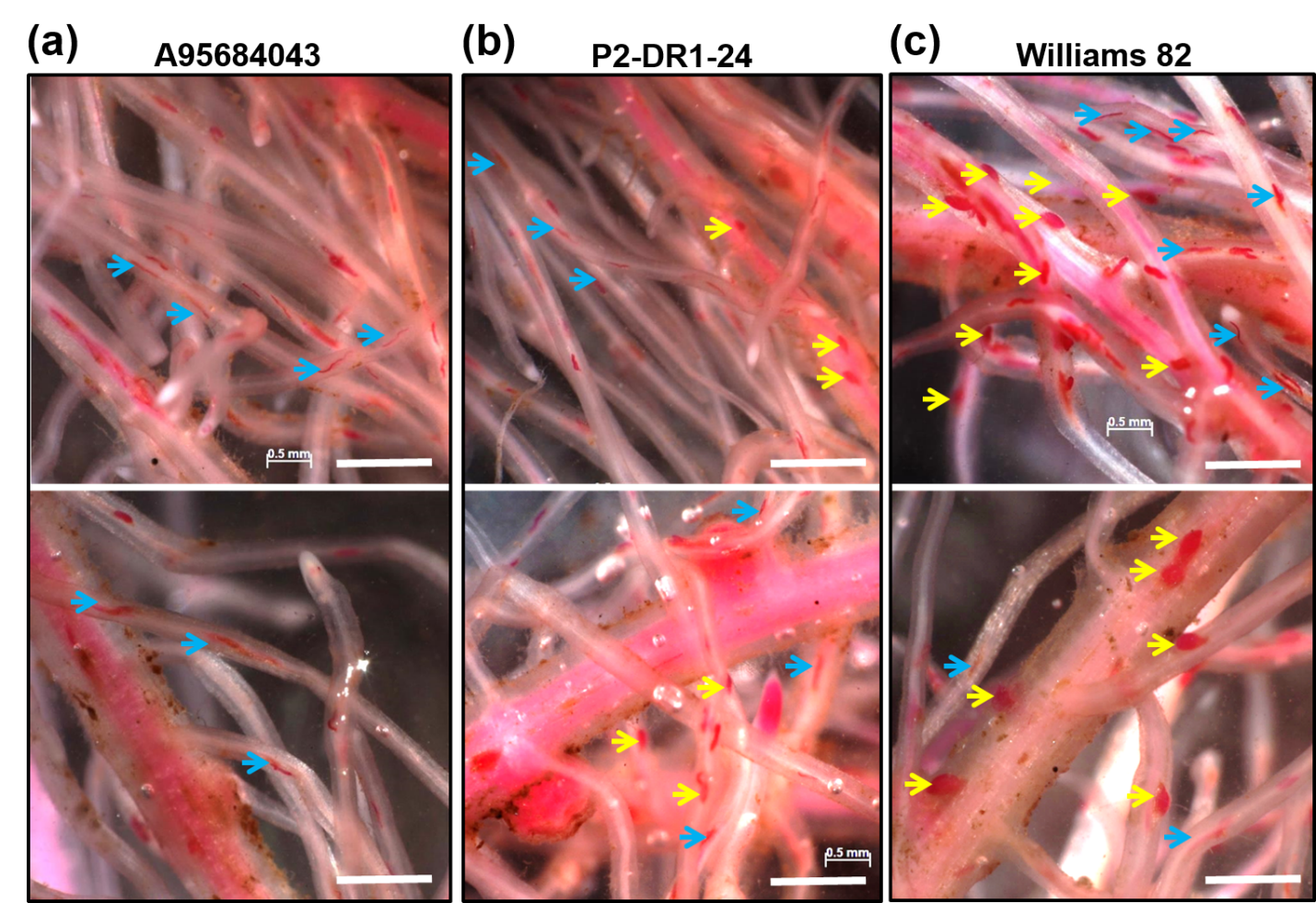


**Figure S9** Responses of transgenic soybean lines overexpressing *GmDR1* to SCN. Acid fuchsine stained roots 15 days following infection with the SCN HG Type 2.5.7. The female adults and juveniles were counted and the data are presented in Figure 4d-e. (a), SCN resistant A95-684043 line; (b), P2-DR1-24 line; (c), SCN susceptible Williams 82 line. Yellow arrows show adult females and blue arrows the juveniles.


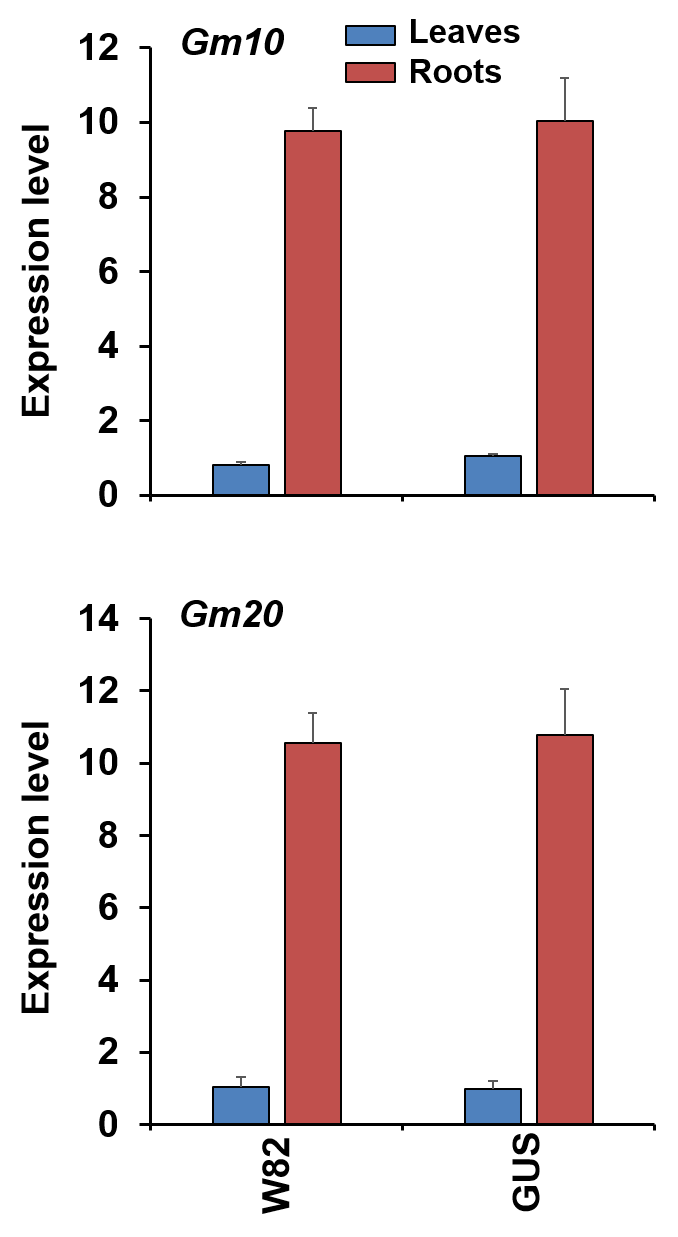


**Figure S10** Expression levels of the genes containing *Promoter 2* and  *Promoter 3*. (a) Expression level of *Gm10* (*promoter 2, Glyma.10g168900).* (b) Expression level of *Gm20* (*promoter 3, Glyma.20g220800*). Data represent mean ± standard error of three independent experiments. Each experiment was comprised of three biological replications, each with expression levels of 6 pooled seedlings for each genotype. Expression values were normalized to the expression levels of the soybean *Elongation factor b* (*ELF1b*) gene (*Glyma.02g44460*) in respective samples. W82, Williams 82. GUS, a transgenic line harboring the *GUS* gene (*GUS*).


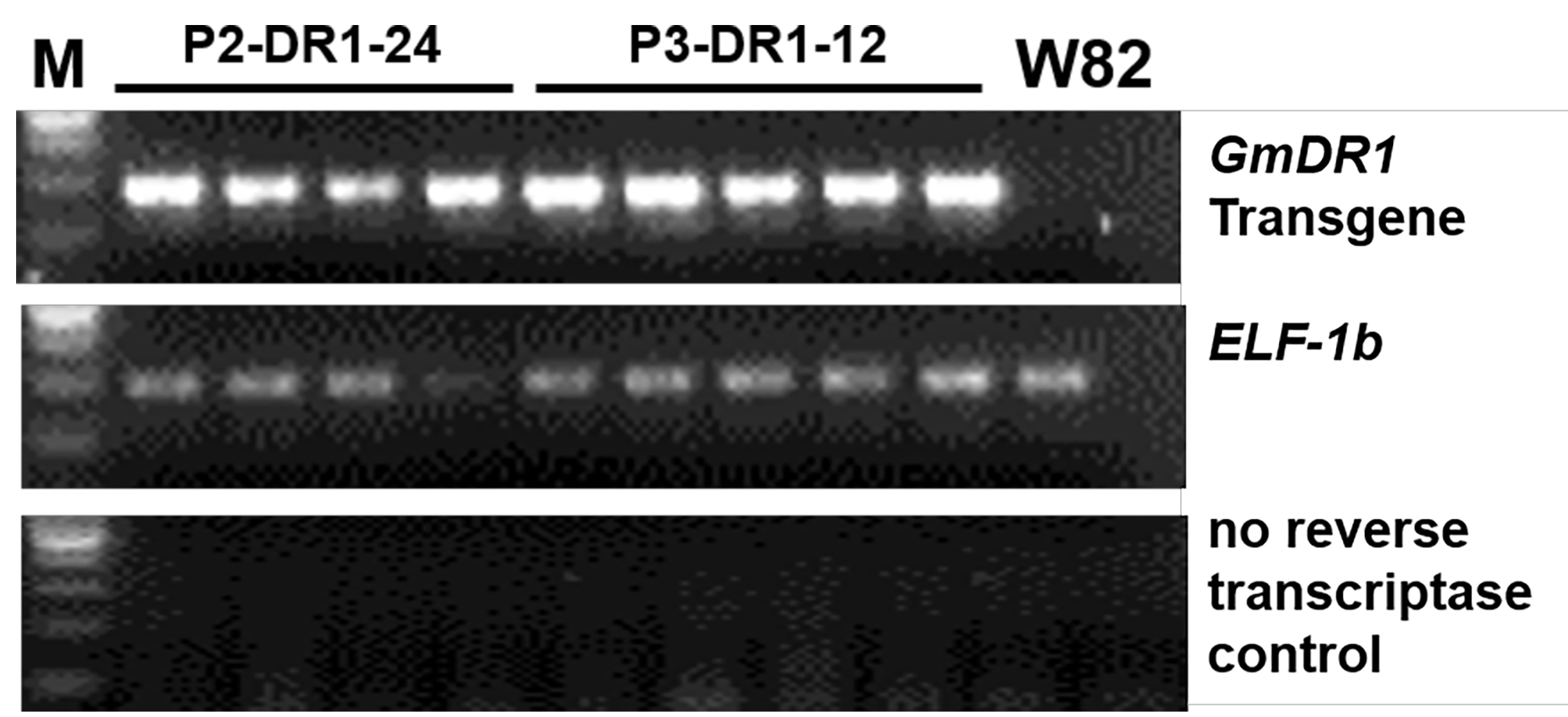


**Figure S11** Expression of *GmDR1* transgenes in leaves of transgenic soybean plants. Total RNAs from the leaves of transgenic lines carrying promoter 2 (P2) and promoter 3 (P3) fused *GmDR1* transgenes and non-transgenic Williams 82 (W82) were investigated for expression of the fusion genes. The following primers were used to conduct the RT-PCR of the two *GmDR1* transgenes: (i) GmDR1-Fw 5’- ctgtggaaatggcaccttatggta -3’and (ii) 35S polyA-Rev 5’- gtagccgacgatggtgcgcc -3’. M, 100 bp marker. W82, RNAs from non-transgenic Williams 82 leaves.

**
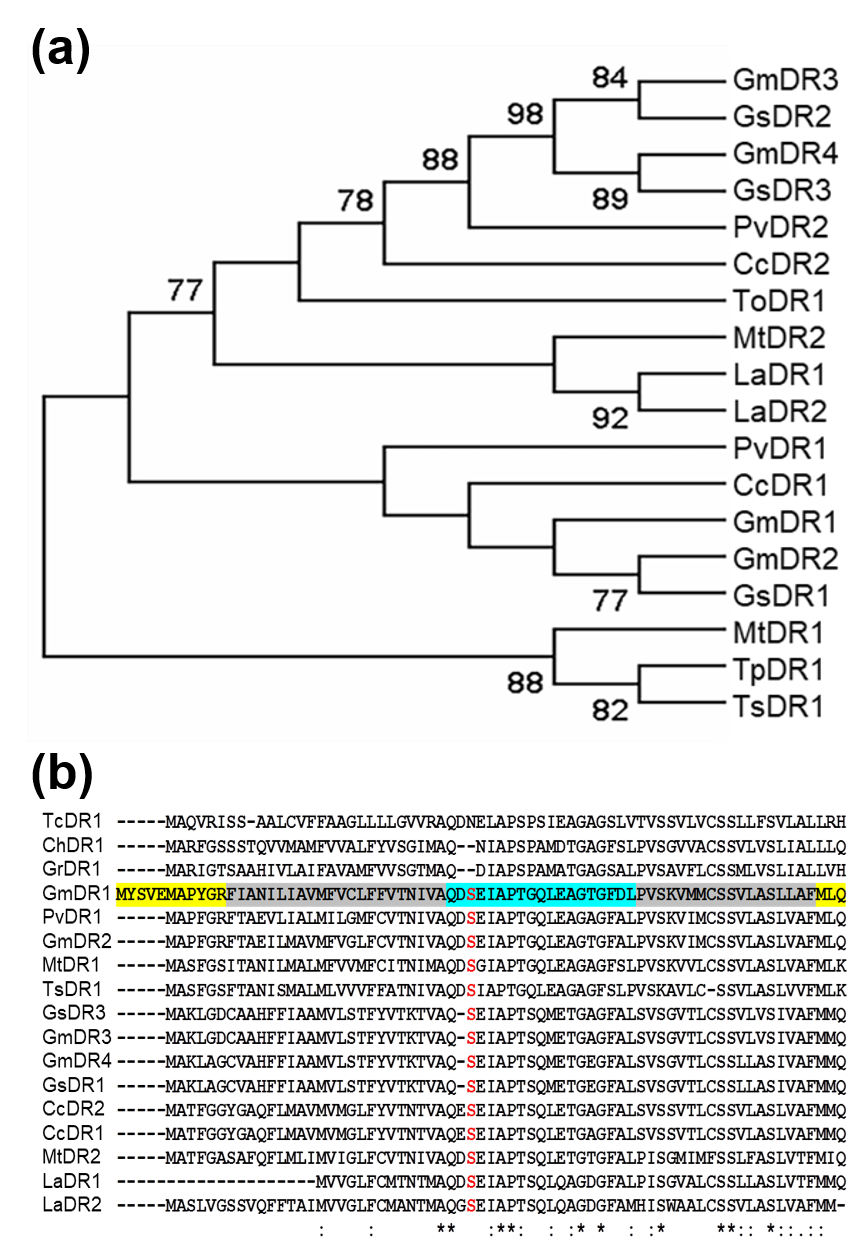
**

**Figure S12** Phylogenetic tree and alignment of *GmDR1* with its closely related homo- and homeologues. (a) Phylogenetic tree of the GmDR1 and GmDR1-like proteins. Numbers represent support values of 1,000 bootstrap replicates. DR, Disease Resistance; Gm, *Glycine max*; Gs, *Glycine soja*, Cc, *Cajanus cajan*; To, *Trema* orientale; Mt, *Medicago truncatula*; La, *Lupinus angustifolius*; Tp, *Trifolium pratense*; Pv, *Phaseolus vulgaris*; Tc, *Theobroma cacao*; Gr, *Gossypium raimondii*; Ch, *Corchorus capsularis*. DR, Disease Resistance; Gm, *Glycine max*; Gs, *Glycine soja*; La, *Lupinus angustifolius*; Ts, *Trifolium subterraneum*; Pv, *Phaseolus vulgaris*; Tc, *Theobroma cacao*; Gr, *Gossypium raimondii*; Ch, *Corchorus capsularis*. (b) Alignment of GmDR1 and its closely related proteins from the GmDR1 clade. The adjacent aspartic acid and serine residues (in red font) are predicted to be involved in protease cleavage and phosphorylation, respectively. Yellow highlight, Cytoplasmic (cyt) domains at N- and C-temini. Gray highlight, transmembrane domains. Blue highlight, non-cytoplasmic or ecto domain; *, conserved residue; :, conservation between groups of amino acids with strong similar properties and ., conservation between groups of amino acids with weak similar properties.

**
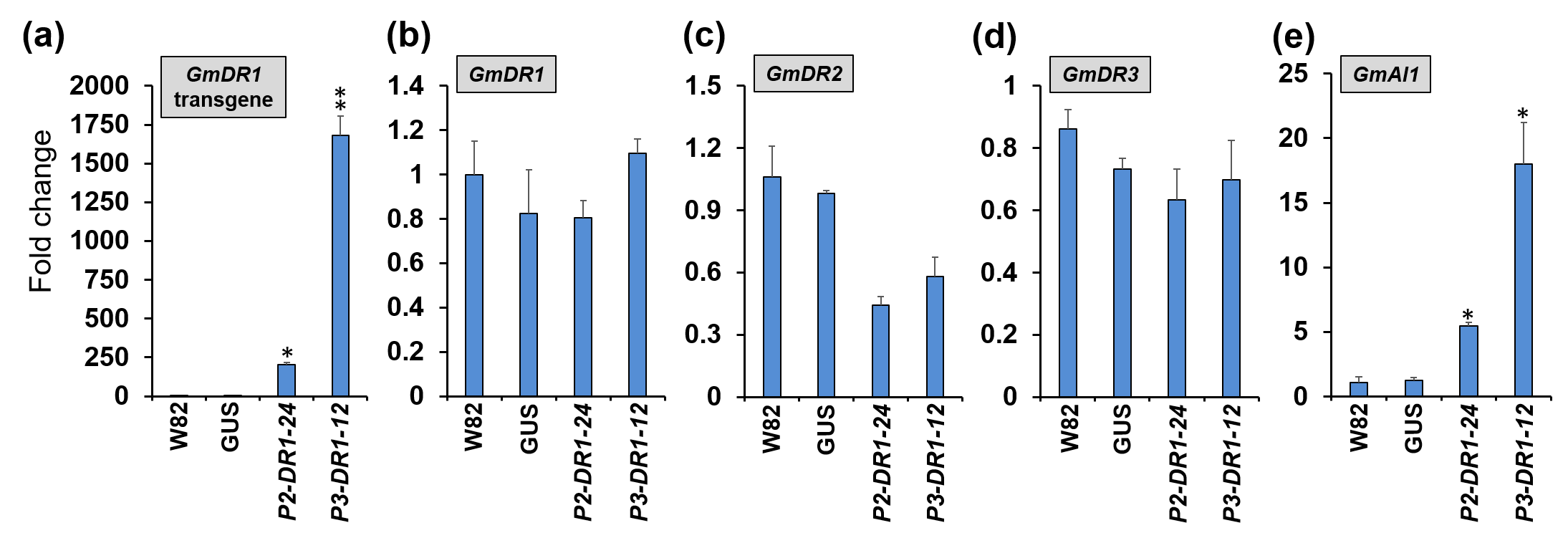
**

**Figure S13** Infleunce of overexpressed *GmDR1* on gene expression in leaves of transgenic soybean lines. Relative transcript abundance of (a) *GmDR1* transgene, (b) *GmDR1* endogenous gene, (c) *GmDR2* (*Glyma.02g180500.1*), (d) *GmDR3 (Glyma.03g139900.1)*, (e) *GmAI1* (*Glyma.06g135900*) in soybean leaves. Data represent mean ± standard error of three independent experiments. In each experiment, three pools of six seedlings for each genotype was considered. Expression values were normalized to the expression levels of the soybean *Elongation factor b* (*ELF1-b*; *Glyma.02g44460*) in respective samples. *, significantly different from control W82. One star, *p* < 0.05; two stars, *p* ≤ 0.001. P2, and P3 are promoter 2 and promoter 3 (Table S1), respectively. W82, Williams 82. GUS, a transgenic line harboring the *GUS* transgene.


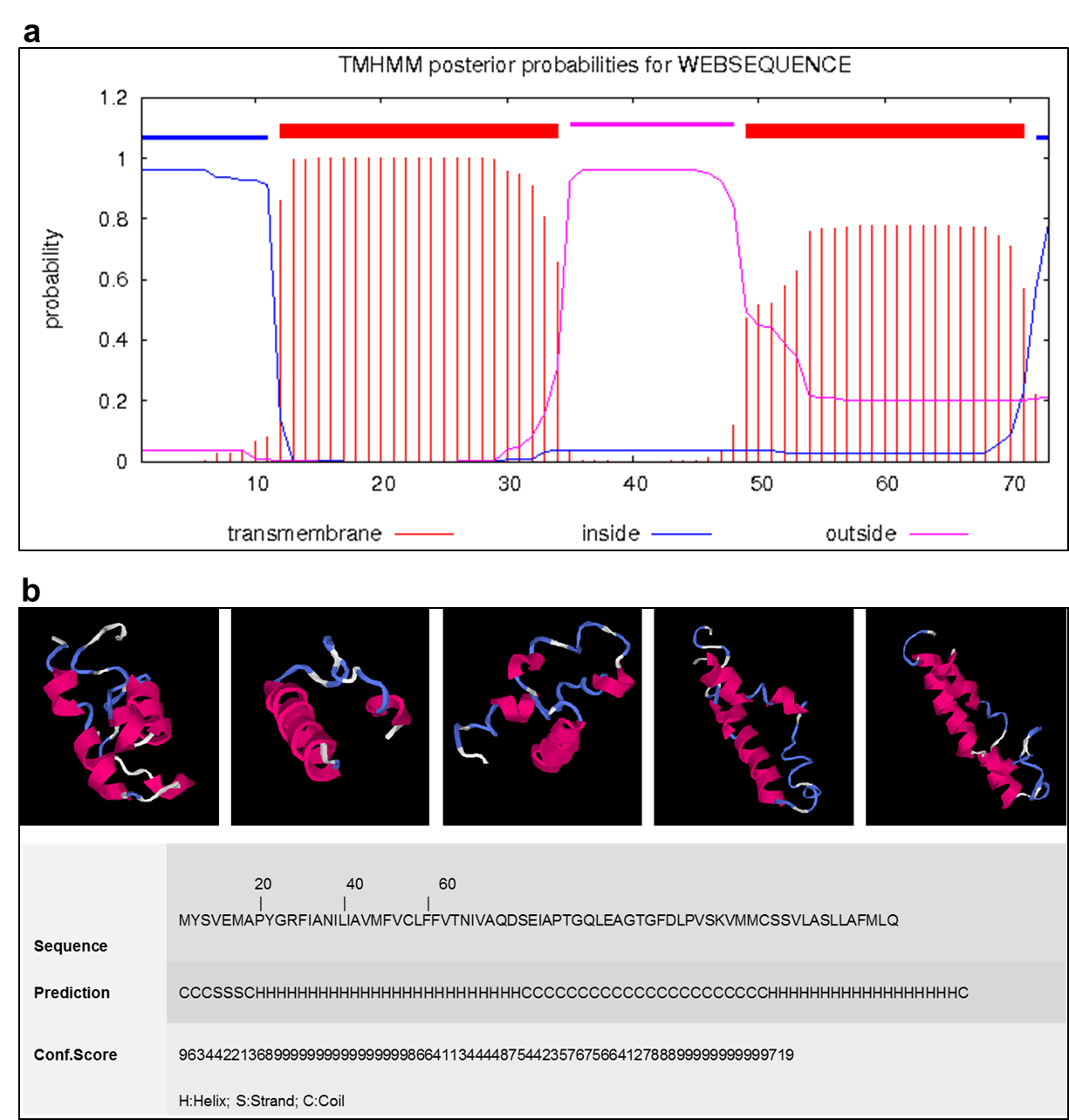

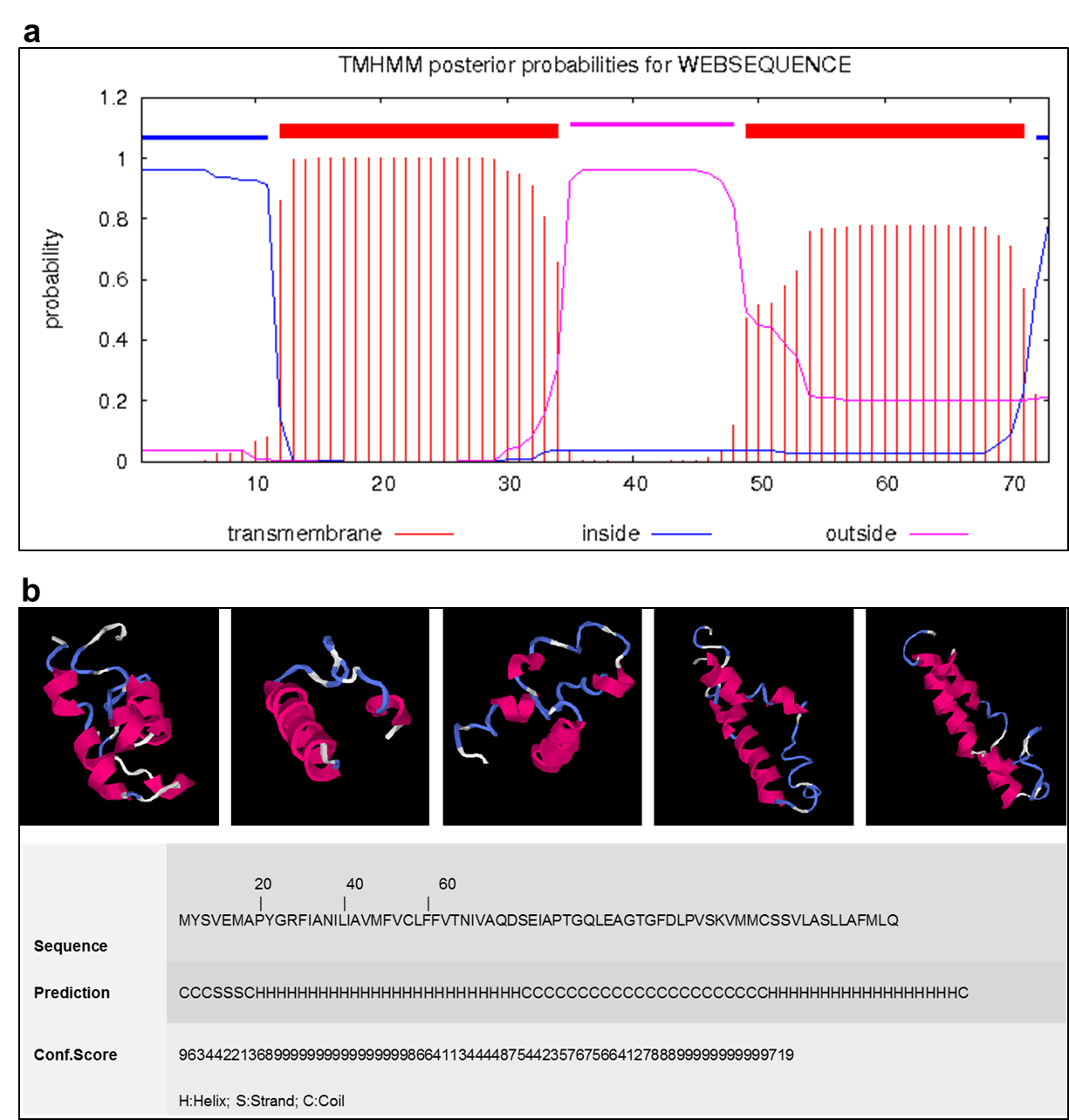


**(b)**

**(a)**

**Figure S14** Putative structure of GmDR1. (a) TMHMM Server v. 2.0 predicted transmembrane helices of GmDR1. (b) 3D models generated for GmDR1 (Predicted using T-Tassel).

**
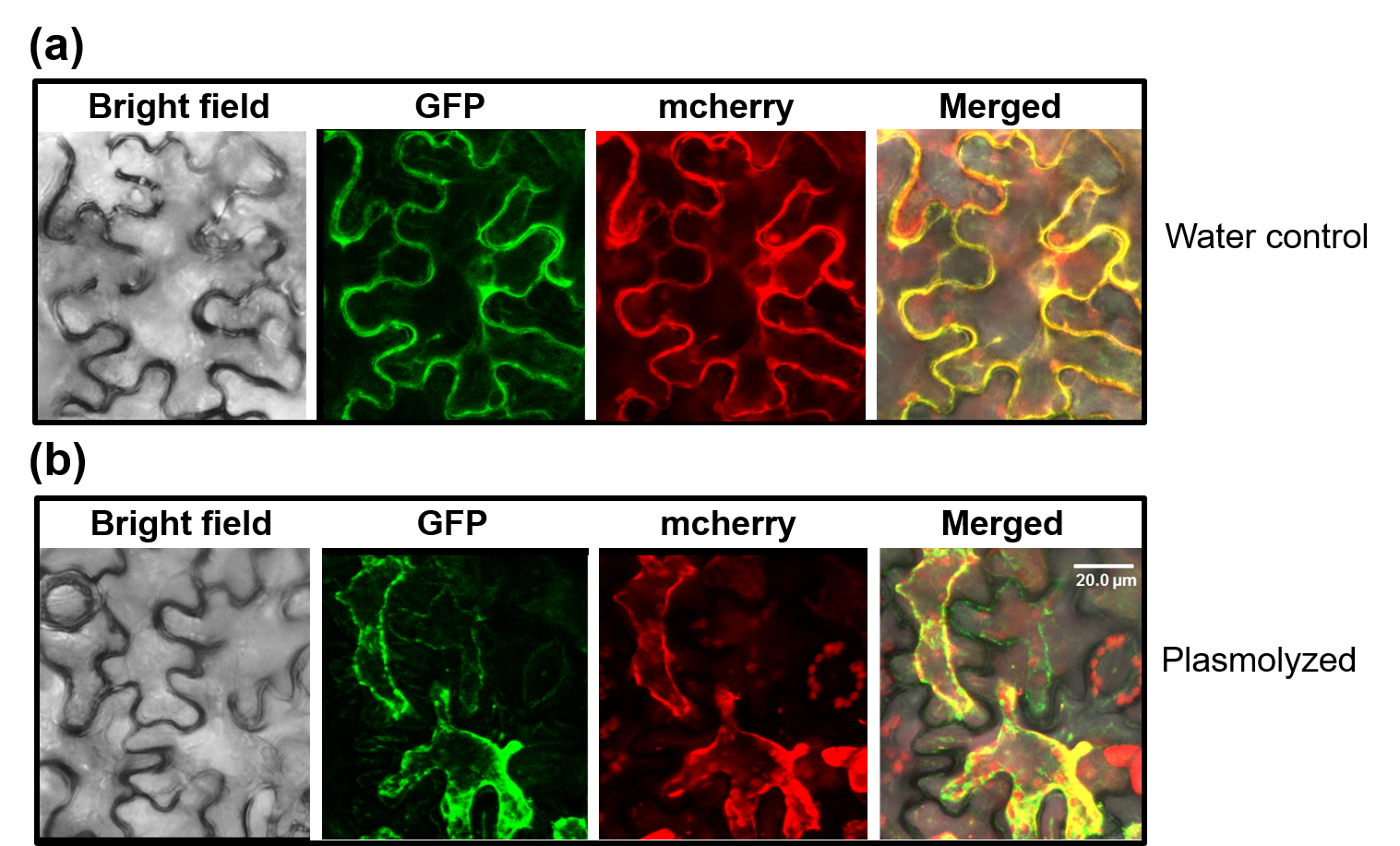
**

**Figure S15** Sub-cellular localization of GmDR1. Transient expression of green fluorescence protein (GFP) fused at the N-terminus of GmDR1 (GFP-GmDR1) and plasma membrane (PM) marker AtPIP2A protein tagged to mCherry (AtPIP2A-MCH) in *Nicotiana benthamiana* leaf epidermal cells. Confocal microscopy was conducted 72 h following transient co-expression of GFP-GmDR1 and AtPIP2A-MCH proteins. (a) Leaf treated with water droplets for 5 minutes. (b) Leaf treated with 5M NaCl droplets for 5 minutes. The peripheral distribution of orange color indicates co-localization of the two transiently expressed green and red fluorescence fusion proteins in the plasma membrane.

**Table S1** Description of the three promoters used in generating the *GmDR1* fusion genes.

| **Promoter**  **name** | **Glycine max Wm82.a1.v1** | **Glycine max Wm82.a2.v1** | **Name** | **Expression in normal condition^1^** |
| --- | --- | --- | --- | --- |
| Promoter 1  (P1 or Prom 1) | *Glyma18g47390* | *Glyma.18g239000* | S-adenosyl-l methionine:carboxyl methyltransferase | Highly expressed in flowers and leaves; low expression in roots |
| Promoter 2  (P2 or Prom 2) | *Glyma10g31210* | *Glyma.10g168900* | Germin-like protein subfamily 1 member 10-related, cupin domain | Highly expressed in roots and nodules |
| Promoter 3  (P3 or Prom 3) | *Glyma20g36300* | *Glyma.20g220800* | Germin-like protein subfamily 1 member 10-related, cupin domain | Highly expressed in roots, low in nodules and leaves |

^1^Expression data from Phytozome V12.1 ([www.phytozome.net/](http://www.phytozome.net/))

**Table S2** Expression levels of three soybean genes in soybean roots following *F. virguliforme* infection (Ngaki *et al*., 2016; Sahu *et al*., 2017).

| **Gene ID**  **(Promoter Name)** | **3-5 days**  **Water treatment** | **10-24 days**  **Water treatment** | **3-5 days**  ***F. virguliforme* infection** | **10-24 days**  ***F.* *virguliforme* infection** |
| --- | --- | --- | --- | --- |
| *Glyma.18g239000*  (Promoter 1) | 0^1^ | 0 | 44 | 126 |
| *Glyma.10g168900*  (Promoter 2) | 802 | 421 | 4,163 | 6,181 |
| *Glyma.20g220800*  (Promoter 3) | 2,442 | 1,274 | 5,362 | 5,065 |

^1^Numbers in the table are Fragments Per Kilobase of transcript per Million mapped reads (FPKM).

**Table S3**  *GmDR1* homo- and homeologoues.

| **Organism** | **Locus ID** | **Gene family** | **Description** | **Identity (%)**^1^ |
| --- | --- | --- | --- | --- |
| *Glycine max* | Glyma.02g180500.1 (*GmDR2*) | Fabidae | Unknown | 86.30 |
| *Phaseolus vulgaris* | Phvul.007g255700.1 | Fabidae | Unknown | 83.60 |
| *Medicago truncatula* | Medtr7g092340.1 | Fabidae | Transmembrane protein, putative | 69.90 |
| *Phaseolus vulgaris* | Phvul.001g136900.1 | Fabidae | Unknown | 68.50 |
| *Glycine max* | Glyma.03g139900.1  (*GmDR3*) | Fabidae | Unknown | 65.80 |
| *Glycine max* | Glyma.19g142700.1  (*GmDR4*) | Fabidae | Unknown | 64.40 |
| *Gossypium raimondii* | Gorai.006g138900.1 | Rosaceae | Unknown | 61.60 |
| *Populus trichocarpa* | Potri.016g052100.1 | Rosaceae | Unknown | 64.40 |
| *Solanum lycopersicum* | Solyc10g080380.1.1 | Penetration-resistance | Unknown | 64.40 |
| *Citrus clementina* | Ciclev10007195m | Rosaceae | Unknown | 61.60 |
| *Citrus sinensis* | orange1.1g047745m | Rosaceae | Unknown | 61.60 |
| *Medicago truncatula* | Medtr1g052125.1 | Fabidae | Transmembrane protein, putative | 57.50 |
| *Theobroma cacao* | Thecc1Eg024815t1 | Rosaceae | Unknown | 52.10 |
| *Prunus persica* | Prupe.7g068300.1 | Fabidae | Unknown | 50.70 |

^1^Identity (%) indicates the percent identity at the amino acid level.

**Table S4** *GmDR1*-co-expressed genes with Pearson correlation coefficient ≥ 0.9 (www. phytozome.jgi.doe.gov).

| **Locus ID** | **Gene name** | **^1^*r*** | **GO - Molecular function** | **GO - Biological process** | **GO - Cellular component** |
| --- | --- | --- | --- | --- | --- |
| *Glyma.04g063800* | Cellulose synthase a catalytic subunit 8 [udp-forming] | 0.95 | Secondary cell wall formation | Cellulose biosynthetic process, cell wall organization, defense response to bacterium, fungus, osmotic stress, and water deprivation | Cell membrane |
| *Glyma.19g179100* | Beta-1,4-xylosyltransferase irx9-related | 0.93 | Galactosylgalactosylxylosylprotein 3-beta-glucuronosyltransferase activity | Cell wall biogenesis & organization, glucuronoxylan metabolic process, plant-type | Golgi apparatus,  Membrane |
| *Glyma.17g242500* | UDP-glucuronate:xylan alpha-glucuronosyltransferase 2 | 0.93 | Glucuronosyltransferase activity | Cell wall biogenesis/degradation | Golgi apparatus,  Membrane |
| *Glyma.12g206900* | Nac domain containing protein 73 | 0.93 | Transcription factor activity, sequence-specific dna binding | Transcription, transcription regulation, response to water deprivation | Nucleus |
| *Glyma.17g169200* | Protein irregular xylem 15-related | 0.93 | Polysaccharide biosynthesis | Plant-type secondary cell wall biogenesis | Golgi apparatus,  Membrane |
| *Glyma.03g257500* | Cytochrome b561/ferric reductase transmembrane | 0.93 | Stress defense, cell wall modifications, iron metabolism | Oxidation-reduction process | Integral component of membrane |
| *Glyma.13g230800* | Protein trichome birefringence-like 37-related | 0.93 | O-acetyltransferase activity | Cell wall organization or biogenesis | Membrane |
| *Glyma.15g084300* | O-fucosyltransferase-like protein | 0.93 | Glycosyltransferase, transferase | Carbohydrate metabolism,  Fructose metabolism, notch signaling pathway | Endoplasmic reticulum, membrane |
| *Glyma.19g008200* | Genomic dna, chromosome 3, tac clone:k24a2-related | 0.92 | Receptor binding | Multicellular organism development |  |
| *Glyma.03g179900* | Fasciclin-like arabinogalactan protein 11 | 0.921 | Cell surface adhesion protein | Plant-type secondary cell wall biogenesis | Plasma membrane |
| *Glyma.13g228200* | O-fucosyltransferase-like | 0.92 | Glycosyltransferase | Cell adhesion | Endoplasmic reticulum, membrane |
| *Glyma.13g334500* | Nad dependent epimerase/dehydratase | 0.92 | Coenzyme binding | Steroid biosynthetic process |  |
| *Glyma.12g174700* | Solute carrier family 35 | 0.92 | Solute carrier | Triose-phosphate transport | Integral component of membrane |
| *Glyma.06g030900* | Nad dependent epimerase/dehydratase | 0.92 | Coenzyme binding | Carbohydrate metabolic process |  |
| *Glyma.06g065000* | Cellulose synthase a catalytic subunit 8 [udp-forming] | 0.92 | Involved in the secondary cell wall formation. Required for the xylem cell wall thickening | Cellulose biosynthetic process, cell wall organization, defense response to bacterium, fungus, osmotic stress, and water deprivation | Cell membrane |
| *Glyma.04g255800* | Glucuronoxylan 4-o-methyltransferase 2-related | 0.91 | Methyltransferase, transferase | Xylan metabolic process |  |
| *Glyma.04g165000* | Flavin-containing monooxygenase fmo gs-ox-like 8-related | 0.91 | Defense response to fungus; oxidation-reduction process | Monooxygenase, oxidoreductase |  |
| *Glyma.12g118700* | NAC domain containing protein 73 | 0.91 | Transcription factor activity, sequence-specific DNA binding | Transcription, transcription regulation, response to water deprivation | Nucleus |
| *Glyma.19g255800* | Cytochrome b561/ferric reductase transmembrane | 0.91 | Stress defense, cell wall modifications, iron metabolism | Oxidation-reduction process | Integral component of membrane |
| *Glyma.19g033600* | Cobra-like protein 4 | 0.91 | Modulates cellulose assembly | Plant-type secondary cell wall biogenesis | Cell membrane, membrane |
| *Glyma.07g020500* | Glycosyltransferase 14 family member | 0.91 | Acetylglucosaminyltransferase activity | Biosynthesis of polysaccharides and glycoproteins in the plant cell wall | Membrane |
| *Glyma.11g079900* | Ring finger domain-containing | 0.91 | Ligase | Protein ubiquitination | Cytoplasm, nucleus |
| *Glyma.19g180700* | Fasciclin-like arabinogalactan protein 11 | 0.90 | Cell surface adhesion protein | Plant-type secondary cell wall biogenesis | Plasma membrane |
| *Glyma.10g139800* | Germin-like protein subfamily 2 member 1-related | 0.90 | Manganese ion binding, nutrient reservoir activity, oxalate decarboxylase activity, plant defense | Oxalate metabolic process, plasmodesmata-mediated intercellular transport, regulation of root development, response to cold | Apoplast, secreted |
| *Glyma.17g072200* | X-box transcription factor-related | 0.90 | Hypersensitive response, plant defense, transcription, transcription regulation | Cellulose biosynthetic process | Membrane |
| *Glyma.02g123800* | (M=3) pthr31116:sf2 - 3-methyladenine glycosylase | 0.90 | Hydrolase | Dna damage, dna repair | Cytoplasm, nucleus |

^1^*r,* Pearson orrelation coefficient.

**Table S5** Primers used in this study.

| **Name** | **Sequence (5’ to 3’)** | **Purpose** | **Reference** |
| --- | --- | --- | --- |
| BsTXI-Ind-Fw | gaattctctagagcataacgtttcgatcatccacg | clone promoter 1 in ptf102 binary vector | Ngaki et al., 2016 |
| BsTXI-Ind-Rev | cgctctagaccatatatctggcctcttttatttgaaacataaattatga | clone promoter 1 in *ptf102* binary vector | Ngaki et al., 2016 |
| BstXI-Root4-Fw | gaattctctagagcctaagacatggaggggaaaataaatg | clone promoter 2 in *ptf102* binary vector | Ngaki et al., 2016 |
| BstXIRoot4-Rev | gaattctctagaccatatatctggccaccagctttggtgtcattgata | clone promoter 2 in *ptf102* binary vector | Ngaki et al., 2016 |
| BamRoot7-Fw | gaattcggatcccggtcccaacaactagtttgcattc | clone promoter 3 in *ptf102* binary vector | Ngaki et al., 2016 |
| BamRoot7-Rev | gaattcggatccggcgcgccgtctgcacgcttgtcaaaatattag | clone promoter 3 in *ptf102* binary vector | Ngaki et al., 2016 |
| BstXITer-Fw | gaattcccatatatctggggcgcaccatcgtcggctac | reclone 35S terminator in *pTF102* | Ngaki et al., 2016 |
| HindTer-Rev | tgctccaccatgttgaccggcatg | reclone 35S terminator in *pTF102* | Ngaki et al., 2016 |
| GmDR1-Fw | gaattcccatatatctggcattaagataatgtattctgtggaaatggc | clone *GmDR1* into *pTF102* | Ngaki et al., 2016 |
| GmDR1-Rev | gaattcccatatatctgggtcttccaattcactgcaacatg | clone *GmDR1* into *pTF102* | Ngaki et al., 2016 |
| GmDR1-RT-Fw | tgtggaaatggcaccttatg | check expression of *GmDR1* transgenes | This study |
| 35S polyA-Rev | gtagccgacgatggtgcgcc | check expression of *GmDR1* transgenes | This study |
| GmDR1/2-pcr-Fw | ctgtggaaatggcaccttatggta | check expression of *GmDR1*/*GmDR2* | This study |
| GmDR1-q-pcr-Rev | ccccaacaagttcaaatttg | check expression of *GmDR1* | This study |
| GmDR1-endo-Rev | ccaacaagttcaaatttgaa | check expression of *GmDR1* | This study |
| GmDR2-pcr-Rev | ccagaaagtgacaattaatttcaaac | check expression of *GmDR2* | This study |
| GmDR3/4-pcr-Fw | cgtgaccaaaactgtggcacaatc | check expression of *GmDR3/DR4* | This study |
| GmDR3-pcr-Rev | aatttcaacacgtacatgataagacca | check expression of *GmDR3* | This study |
| GmDR4-pcr-Rev | catcaaacatatatataatttcag | checking the expression of *GmDR4* | This study |
| GmDR1-gfp-Fw | cgcggatccatgtattctgtggaaatggcaccttatg | Subcellular localization of GmDR1 | This study |
| GmDR1-gfp-Rev | cgcggatcctcactgcaacatgaatgccaaaag | Subcellular localization of GmDR1 | This study |
| Bar-Fw | ctgccagaaacccacgtcatg | checking the expression of *bar* gene | This study |
| Bar-Rev | ctgcaccatcgtcaaccactaca | checking the expression of *bar* gene | This study |
| ELF1b-Fw | cgctcaaggggtaagattca | checking the expression of *ELF1b* | Ngaki et al., 2016 |
| ELF1b-Rev | cccacaataaaccaggcatc | checking the expression of *ELF1b* | Ngaki et al., 2016 |
| ELF1b-Rev2 | tagcagcctccctttcctctg | checking the expression of *ELF1b* | Ngaki et al., 2016 |
| FvTox1-Fw | gcaggccatgttggttctgta | check the fungal biomasses in roots | Mbogung et al., 2011 |
| FvTox1-Rev | gcacgtaaagtgagtcgtctcatc | check the fungal biomasses in roots | Mbofung et al., 2011 |
| Reference Fw | catatcgccacttctccaccga | check the fungal biomasses in roots | Ngaki et al., 2016 |
| Reference Rev | gcacgtaaagtgagtcgtctcatc | check the fungal biomasses in roots | Ngaki et al., 2016 |
| GmPR1-1-Fw | gggtgaatgagaaatccaagtataactac | qRT-PCR for transcript level | Xu et al., 2016 |
| GmPR1-1-Rev | ggtctttggccaata tag ttg cca gtg | qRT-PCR for transcript level | Xu et al., 2016 |
| GmPR1-2-Fw | agaggcagaggtgggttct | qRT-PCR for transcript level | Xu et al., 2018 |
| GmPR1-2-Rev | tcaccaacaaagttgccagg | qRT-PCR for transcript level | Xu et al., 2018 |
| GmPR2-Fw | gaaacccgaacactcatttg | qRT-PCR for transcript level | Xu et al., 2018 |
| GmPR2-Rev | caccatcaagatatgctcctc | qRT-PCR for transcript level | Xu et al., 2018 |
| GmEDS1a-Fw | atggctggagggttgcttg | qRT-PCR for transcript level | Wang et al., 2014 |
| GmEDS1a-Rev | ccaagatcccgggaagctg | qRT-PCR for transcript level | Wang et al., 2014 |
| GmEDS1b-Fw | atggctggaggatcacttgg | qRT-PCR for transcript level | Wang et al., 2014 |
| GmEDS1b-Rev | gatcccaagatcccgggaag | qRT-PCR for transcript level | Wang et al., 2014 |
| GmJAR1-Fw: | gtggtgaaggttatgggattccac | qRT-PCR for transcript level | This study |
| GmJAR1-Rev: | cctctgctagcaacttccctg | qRT-PCR for transcript level | This study |
| GmNAC6-Fw | ccaacaaaagcacttgtggca | qRT-PCR for transcript level | Pimenta et al., 2016 |
| GmNAC6-Rev | ggactattcaactgagcccaaaag | qRT-PCR for transcript level | Pimenta et al., 2016 |
| GmICS1-Fw | ggccatttcggagctgg | qRT-PCR for transcript level | Lin et al., 2013 |
| GmICS1-Rev | aggagaaggtggttctgtgagaga | qRT-PCR for transcript level | Lin et al., 2013 |
| GmNPR1-1-Fw | ctacagaagcatcattttcaacatctt | qRT-PCR for transcript level | Lin et al., 2013 |
| GmNPR1-1-Rev | ctacagaagcatcattttcaacatctt | qRT-PCR for transcript level | Lin et al., 2013 |
| GmNPR1-2-Fw | caattgaccaagagcttccaa | qRT-PCR for transcript level | Lin et al., 2013 |
| GmNPR1-2-Rev | ctatagaagcatcgttttcaacatctc | qRT-PCR for transcript level | Lin et al., 2013 |
| GmDR1-endo-Rev | ccaacaagttcaaatttgaa | qRT-PCR for transcript level | Lin et al., 2013 |
| Gm06-Fw | gatgtccatcttcagccacttcgag | qRT-PCR for transcript level | Studham & MacIntosh, 2013 |
| Gm06-Rev | ggcgggttttgcttggatccaccgg | qRT-PCR for transcript level | Studham & MacIntosh, 2013 |
| GmACS1k-Fw | cttaggctcagtttctcttcaaggatatttgat | qRT-PCR for transcript level | This study |
| GmACS1k-Rev | cgctcgagtagaacccagatccaatc | qRT-PCR for transcript level | This study |
| P2-Fw | ggttgtttacctcttcgttgtgctgatg | qRT-PCR for transcript level | This study |
| p2-rev | gcataagcaaacttaggatccttgc | qRT-PCR for transcript level | This study |
| P3-Fw | ggtggtctatatggagtgttcgtg | qRT-PCR for transcript level | This study |
| P3-Rev | gtgtgttgagccctaatatttcattaac | qRT-PCR for transcript level | This study |

**Table S6** SDS foliar disease severity scale (modified based on Hartman *et al*., 1997 and Li *et al*., 2009).

| **Disease score** | **Foliar symptoms** |
| --- | --- |
| 1 | no symptoms (0% foliage affected) |
| 2 | slight symptom development with mottling and mosaic on leaves (1-20% foliage affected) |
| 3 | moderate symptom development with interveinal chlorosis and necrosis on foliage (21-50% foliage affected) |
| 4 | heavy symptom development with interveinal chlorosis and necrosis (51-80% foliage affected) |
| 5-7 | severe interveinal chlorosis and necrosis (81-100% foliage affected) |
